# Unraveling the meta-hallmarks between senescent and tumor cells: A new perspective for senolytic drug discovery

**DOI:** 10.1016/j.apsb.2025.08.010

**Published:** 2025-08-19

**Authors:** Wei Liu, Bo Fan, Te Fang, Hongyao Li, Jin Zhang, Bo Liu, Zhiyu Liu

**Affiliations:** a Department of Biotherapy, Cancer Center and State Key Laboratory of Biotherapy, West China Hospital, Sichuan University, Chengdu 610041, China; b Department of Urology, Institute of Precision Drug Innovation and Cancer Center, Second Affiliated Hospital of Dalian Medical University, Dalian 116023, China; c Department of Anesthesiology, the First Affiliated Hospital of China Medical University, Shenyang 110001, China; d School of Pharmacy, Shenzhen University Medical School, Shenzhen University, Shenzhen 518060, China

**Keywords:** Senolytic drug, Cell senescence, Cancer, Meta-hallmark, Drug discovery, Repurposing, Anti-tumor drug, Senolytic therapy

## Abstract

Aging and cancer share overlapping characteristics, referred to as meta-hallmarks, which elucidate the convergent, antagonistic, or contradictory relationships between aging and cancer. Likewise, as a key characteristic of aging, senescent cells share some meta-hallmarks with tumor cells. These hallmarks include apoptosis resistance, metabolic alterations, secretory phenotypes, epigenetic reprogramming, and immune surveillance, all of which play pivotal roles in both tumorigenesis and senescence. Moreover, senolytic drugs, which are a class of agents selectively designed to eliminate senescent cells, have emerged as promising therapeutic agents in oncology and aging-related diseases. Since the discovery of the first senolytic drug in 2015, a diverse array of such agents has been developed. Notably, most senolytic drugs are repurposed from existing anti-tumor therapies, leveraging their shared mechanisms with senescent cells and tumor cells. Thus, this review examines the similarities between senescent cells and tumor cells, providing a better understanding of the meta-hallmarks. Besides, we categorize existing senolytic drugs based upon meta-hallmarks and elucidate the potential molecular mechanisms underlying their effects. By integrating insights from cancer and senescence research, this work aims to inspire innovative strategies for senolytic drug discovery.

## Graphical abstract

This review summarizes the meta-hallmarks between senescent and tumor cells and elucidates the molecular mechanisms of existing senolytic drugs based upon these meta-hallmarks; thereby inspiring the innovative strategies for senolytic drug discovery.

**Figure:**
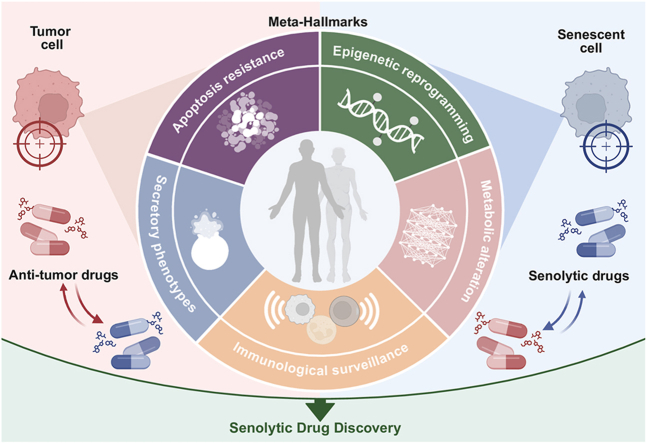

## 1 Introduction

Aging refers to an inevitable and natural process of biological organisms undergoing gradual changes over time. This process includes the progressive decline in the function of cells, tissues, and organs, ultimately leading to the death of the organism^1^. Aging is also characterized by a decline in metabolic and functional capacities of the organism, predisposing individuals to various diseases, including neurodegenerative diseases, cardiovascular diseases, metabolic dysfunctions, immune system deficiencies, and cancer^2^. Among these, cancer stands out as a leading cause of mortality, with aging recognized as its most significant risk factor. The majority of cancer cases occur in older populations, underscoring the critical interplay between aging and tumorigenesis. In 2023, Professor Guido Kroemer identified the 12 hallmarks of aging, including genomic instability, telomere attrition, epigenetic alterations, loss of proteostasis, disabled macroautophagy, deregulated nutrient-sensing, mitochondrial dysfunction, stem cell exhaustion, altered intercellular communication, chronic inflammation, and cellular senescence^3^. Recently, Professor Guido Kroemer identified extracellular matrix changes and psychosocial isolation as additional hallmarks of aging^4^. Among them, cellular senescence stands as a critical and distinctive hallmark of aging. Cellular senescence (also referred to as senescence) is typically used to describe the aging process at the cellular level, and senescent cells are characterized by irreversible cell cycle arrest in response to stressors such as DNA damage, oxidative stress, or oncogene activation^5^, where cells cease to divide but do not undergo apoptosis^6^^,^^7^.

Over recent decades, the interplay between senescence and cancer has garnered significant research interest. Cellular senescence plays a dual role in cancer biology, acting as both a tumor suppressor and a promoter of tumorigenesis^8^. On one hand, senescence serves as a critical barrier against cancer development by inducing irreversible cell cycle arrest in response to oncogenic stimuli, DNA damage, or oxidative stress^9^. This process is primarily mediated by the activation of tumor suppressor pathways, such as p53/p21 and p16/RB, which halt the proliferation of potentially malignant cells^10^. Additionally, senescence-associated secretory phenotypes (SASP) can reinforce this anti-tumor effect by recruiting immune cells to eliminate senescent cells, thereby preventing tumor progression^11^. On the other hand, senescent cells can also contribute to tumorigenesis through the secretion of SASP factors, which include pro-inflammatory cytokines, growth factors, and matrix metalloproteinases. These factors can create a tumor-promoting microenvironment by inducing epithelial-to-mesenchymal transition (EMT), angiogenesis, and immune evasion^12^. Moreover, the persistence of senescent cells in tissues can lead to chronic inflammation and genomic instability, further driving cancer progression. The balance between the tumor-suppressive and tumor-promoting effects of senescence is influenced by various factors, including the cellular context, the nature of the senescence-inducing stimuli, and the efficiency of immune-mediated clearance of senescent cells^13^. Understanding the complex role of senescence in cancer is crucial for developing therapeutic strategies that harness its tumor-suppressive potential while mitigating its pro-tumorigenic effects. Targeting senescence pathways, such as SASP modulation or senolytic therapies, offers promising avenues for cancer treatment, particularly in combination with conventional therapies to enhance efficacy and reduce resistance.

In 2023, Professor Guido Kroemer summarized the meta-hallmarks of aging and cancer, such as genomic instability, chronic inflammation, dysbiosis, and epigenetic alterations^14^. Likewise, as a key characteristic of aging, senescent cells share similar traits with tumor cells, which are summarized in this review, including resistance to apoptosis, metabolic reprogramming, specific secretory phenotypes, epigenetic modifications, and immune surveillance^6^^,^^15^, ^16^, ^17^, ^18^. These meta-hallmarks provide promising avenues for developing therapeutic strategies targeting senescence and its connection to cancer. It should be emphasized that senescent cells and tumor cells are not two distinct cell types, and senescence can indeed occur within tumor cells. For instance, tumor cells often undergo therapy-induced senescence following radiotherapy or chemotherapy^11^. In this manuscript, we focus on two distinct yet interrelated biological states—cellular senescence and tumorigenesis—centering our analysis on senescent cells and tumor cells as biological entities, with a systematic investigation of the similarities between these two cell states.

Senolytic drugs, which selectively eliminate senescent cells while sparing normal ones, show significant potential for treating age-related diseases^19^. The first-generation senolytic drugs, dasatinib (D) and quercetin (Q), were discovered in 2015 using a hypothesis-driven, bioinformatics-informed approach^19^. These drugs have demonstrated promising results in preclinical studies and are currently undergoing clinical trials for various diseases^19^. Recent advancements, such as single-cell screening, big data, and high-performance computing, have accelerated the discovery of novel senolytic agents. These methodologies have led to the identification of various senolytic drugs such as Bcl-2 family protein inhibitors, heat shock protein inhibitors, BET family protein inhibitors, and p53^20^. Notably, many senolytic drugs have been repurposed from anti-tumor therapeutics, with their mechanisms of action closely linked to the meta-hallmarks of cellular senescence and cancer^21^. For instance, panobinostat, a histone deacetylase (HDAC) inhibitor with epigenetic activity and FDA-approved for the treatment of multiple myeloma, has been demonstrated to function as a senolytic agent by increasing histone 3 (H3) acetylation and eliminating chemotherapy-induced senescent cells in non-small cell lung cancer and head and neck squamous cell carcinoma^22^; similarly, ABT737, a Bcl-2 family inhibitor knowing for inducing apoptosis in gastric cancer and breast tumor cells^23^, has shown considerable potential in clearing senescent macrophages and neuronal cells, thereby ameliorating aging-related diseases^24^^,^^25^. These examples highlight a novel strategy for senolytic drug discovery by repositioning anti-tumor drugs or tumor-related drugs as senolytic drugs.

In this review, we systematically summarize the history and development of senolytic drugs and their applications in various aging-related diseases. Moreover, we focus on the more microscopic and specific aspects of aging and cancer, with particular emphasis on senescent cells and tumor cells, and summarize the meta-hallmarks of senescent cells and tumor cells, emphasizing how most existing senolytic drugs are derived from anti-tumor agents. This approach suggests a promising avenue for repurposing antineoplastic drugs as senolytic drugs or adopting similar strategies to develop senolytic drugs with antineoplastic drugs, thus providing new clues for the development of senolytic drugs. Finally, we also discuss the current landscape and challenges in senolytic drug discovery, along with future directions, highlighting the role of senolytic drugs in senescence and the application of innovative methods and cutting-edge technologies in the development of these therapeutics.

## 2 The history and development of senolytic drugs

Cell senescence refers to the state of permanent arrest of proliferation, which was identified in 1961 by Hayflick and Moorehead through studies on human fibroblast cultures^26^. Until 1995, the discovery of the senescence-associated *β*-galactosidase biomarker promoted the understanding of senescence *in vivo*^27^. In 2004, research demonstrated a negative correlation between senescent cell burden and lifespan, which inspired the development of new drugs to remove senescent cells and thus extend lifespan. A pivotal milestone came in 2011 with the establishment of the *INK-ATTAC* transgenic mouse model, which was established to clear highly p16-expressing cells of progeroid mice, thus improving aging-related phenotypes^28^^,^^29^. In 2013, the first demonstration of selective pharmacological elimination of senescent cells by synthetic lethal metabolic pathways was reported^30^. This breakthrough was followed in 2015 by Dr. James Kirkland's introduction of the term “senolytics” and the discovery of the first-generation senolytic drugs, dasatinib and quercetin, using a hypothesis-driven, bioinformatics-informed approach^31^. Subsequently, multiple senolytics, such as navitoclax (2016)^32^, 17-DMAG (2017)^33^, fisetin (2018)^34^, EF24 (2019)^35^, Cardiac Glycosides (2019) ^36^ were screened based on this method. Interestingly, most of these drugs achieve the elimination of senescent cells mainly by targeting senescent cell antiapoptotic pathways (SCAPs) and inducing apoptosis.

The development of second-generation senolytic drugs marked a significant evolution. These drugs were discovered based on the high-throughput compound library screens and other traditional drug discovery methods. Compared with the first-generation drugs, these drugs show a wealth of mechanisms to clear senescent cells, such as regulating autophagy (ARV-825)^37^, metabolism (aminooxyacetic acid)^38^, immune (azithromycin)^39^, and pyroptosis (nigericin)^40^. Moreover, the exploitation of delivery systems such as nano-particles further improves the therapeutic and diagnostic applications of senolytic drugs^41^.

It is worth noting that the therapeutic potential of some senolytic drugs has been demonstrated in clinical trials. The most widely studied is a group of dasatinib and quercetin, which has been verified in various aging-related diseases, such as Idiopathic pulmonary fibrosis (NCT028749819), diabetic chronic kidney disease (NCT02848131), and Alzheimer's disease (NCT0463124). Other drugs, such as UBX0101, were exploited for the treatment of osteoarthritis (NCT04229225), and fisetin was evaluated for age-related osteoporosis (NCT04313634). While these studies highlight the transformative potential of senolytic drugs, the field is still in its infancy, requiring deeper and more systematic research to realize their full potential.

## 3 The therapeutic effects of senolytic drugs in various diseases

Since their discovery, senolytic drugs have demonstrated significant anti-aging effects across multiple disease models, emerging as a promising therapeutic strategy. Beyond the clinical trials previously mentioned, numerous senolytic drugs have shown efficacy in preclinical studies targeting aging-related diseases.

In the cardiovascular system, the local delivery of ABT263 effectively reduced the expression of pro-inflammatory molecules and matrix-degrading proteases, attenuating adverse cardiac remodeling and exhibiting therapeutic effects on ischemia- and reperfusion-injured myocardium^42^. Additionally, ABT263 selectively decreased IL-6 and senescent vascular smooth muscle cells, substantially reducing atherogenesis^43^. Another senolytic drug, RG-7112, demonstrated the ability to mitigate vascular calcification^44^.

In the skeletal system, prolonged combination therapy with dasatinib and quercetin (D + Q) improved age-related disc degeneration in mice and significantly ameliorated postmenopausal osteoporosis while restoring mesenchymal stem cell function^45^^,^^46^. Similarly, UBX0101 rescued protein oxidative stress in knee joints, offering a potential treatment for osteoarthritis^47^. Fisetin, a natural senolytic agent, also holds promise for osteoarthritis treatment due to its capacity to alleviate IL-1*β*-stimulated chondrocyte inflammation, extracellular matrix degradation, cartilage apoptosis, and deleterious aging-associated phenotypes^48^.

Senolytic drugs have been extensively studied in neurodegenerative diseases. For example, the D + Q combination showed good tolerability and modulation of senescence-related biomarkers in a phase I clinical trial for Alzheimer's disease, supporting its feasibility as a therapeutic approach^49^. AP20187 reduced microglial activation, suppressed SASP factor expression, and improved cognitive function in aged mice^50^. ABT-263 has also been reported to decrease pro-inflammatory microglia and macrophages, promoting neuronal survival and offering a potential therapeutic strategy for multiple sclerosis^51^.

Senolytic drugs also exhibit potential in managing metabolism-related diseases. The D + Q combination reduced hepatic steatosis by eliminating senescent liver cells incapable of metabolizing fatty acids, supporting its use in treating non-alcoholic fatty liver disease^52^. Furthermore, these drugs improved metabolism by reducing adipose tissue inflammation^53^. In diabetes, D + Q modified insulin resistance^54^, while UBX1325 improved hyperglycemia-induced cellular senescence and inflammation in vascular endothelial cells, representing an effective intervention for diabetic macular edema^55^. Additionally, UBX1967 decreased pathological angiogenesis associated with aging and promoted physiological vascular repair, offering a therapeutic approach for diseases like diabetic retinopathy^56^.

Cancer, another category of aging-related diseases, is often associated with cellular senescence induced by oncogene activation, oncogene loss, or conventional chemotherapy and radiotherapy, etc. Persistent senescent tumor cells can pose risks to tumor progression; thus, targeting and eliminating these cells with senolytic drugs has emerged as a promising strategy for cancer therapy. Correspondingly, these senescent cells also have unique alterations in cellular processes, such as dysregulation of the cell cycle and apoptosis, which make senescent cells susceptible. These specific vulnerabilities can be targeted by senolytic drugs. This has also inspired a novel therapeutic option for cancer, a sequential treatment with a first drug to induce senescence and a second drug to selectively kill senescent cells. For example, the commonly used radiotherapy and chemotherapy have their oncologic effects as well as senescence-inducing effects. The sequential use of senolytic drugs allows for better therapeutic performance. For instance, ABT-263 has entered clinical trials to synergize with various chemotherapeutic agents for treating multiple solid tumors. Further study suggested that ABT-263 can effectively clear senescent bone marrow hematopoietic stem cells and muscle stem cells from sublethally irradiated or normally aged mice^57^. Similarly, ABT-737 demonstrated synergistic therapeutic effects with chemotherapy and radiotherapy in small-cell lung and breast cancers, with recent reports highlighting its combined cytotoxicity with an AKT inhibitor^58^. ABT-737 can also decrease tumor burden and ameliorate tumorigenesis in KRAS-driven lung cancer models by clearing senescent tumorigenesis^25^. Additionally, ARV-825 provokes senolysis to inhibit hepatocellular carcinoma progression and enhance chemotherapeutic efficacy *via* the autophagy pathway^37^.

**Figure 1:**
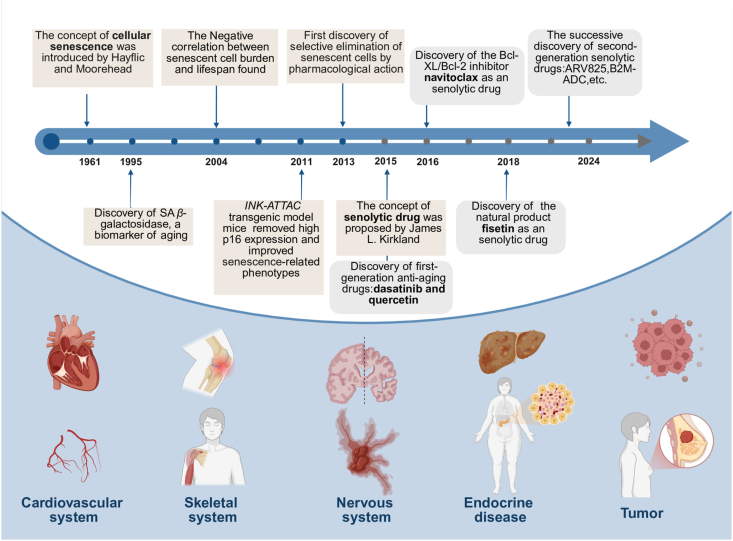
The representative history and development of senolytic drugs and their therapeutic effects in various diseases.

In summary, senolytic drugs have widespread applications in age-related diseases, including cardiovascular, skeletal, neurological, metabolic disorders, and cancer, underscoring their potential as transformative therapeutic agents (Fig. 1).

## 4 The meta-hallmarks between senescent cells and tumor cells

Given the critical role of senolytic drugs in age-related diseases, the development of entirely new senolytic drugs holds immense significance. Interestingly, the majority of existing senolytic drugs are repurposed from existing anti-tumor agents, suggesting a potential relationship between aging and cancer. In 2013, Professor Guido Kroemer identified the 9 hallmarks of aging, which were further refined into 14 hallmarks in 2025^3^^,^^59^. He also summarized the meta-hallmarks of aging and cancer, and elucidated their complex interrelationship^14^. Senescent cells and tumor cells are also characterized by a series of overlapped meta-hallmarks, which are closely related to the molecular mechanism of senolytic drugs to clear senescent cells. Here, we focus on senescent cells (rather than aging) *versus* tumor cells, and summarize their meta-hallmarks such as apoptosis resistance, metabolic alterations, secretory phenotypes, epigenetic reprogramming, and immune surveillance to guide the discovery of senolytic drugs.

### 4.1 The apoptosis resistance of senescent cells and tumor cells

Apoptosis is a mode of programmed cell death characterized by a complex series of biochemical pathways that are essential for development, tissue repair, and the elimination of damaged or virus-infected cells. Typically, apoptosis contributes to the maintenance of a healthy organism by facilitating proper cellular turnover and growth. However, apoptosis resistance in senescent cells refers to their inability to undergo programmed cell death due to defects in the apoptotic machinery. This phenomenon is particularly significant in tumors, as it allows tumor cells to survive and proliferate in hostile environments, thereby promoting tumor progression and metastasis^60^. The mechanisms underlying this resistance stem from the unique regulation of the cell cycle and anti-apoptotic pathways in senescent cells and tumor cells, alongside other related mechanisms^61^.

#### 4.1.1 Disturbed p53 network

In senescent cells, there is an increased accumulation of DNA damage coupled with a diminished capacity for DNA repair. The reduced sensitivity of these cells to DNA damage response signaling enables senescent cells to evade apoptosis even in the presence of extensive DNA damage. This imbalance between DNA repair and damage facilitates the persistence of senescent cells, despite ongoing genomic instability. In addition, the p53 network in senescent cells becomes dysregulated, characterized by the overexpression of p53, p21, and cyclin-dependent kinase inhibitors. This hyperactivity may accelerate the aging process. Concurrently, the functionality of p53 declines with age, impairing its ability to initiate apoptosis^62^. Several studies have demonstrated that, under senescence-inducing conditions, p53 mutant mice exhibit reduced apoptosis compared to wild-type mice^63^. Furthermore, the loss of p53 function negatively affects glycolytic metabolism and activates IKK*β*, which subsequently stimulates the NF-*κ*B pathway, contributing to apoptosis resistance^64^. In summary, the dysregulation of the p53 network plays a crucial role in promoting apoptosis resistance in senescent cells.

Conversely, p53 serves as a crucial tumor suppressor that inhibits cancer development by inducing cell cycle arrest and apoptosis, primarily through the transcriptional activation of genes involved in these processes. When the *TP53* gene, which encodes p53, is inactivated or its expression is diminished, tumor cells can evade apoptosis. Mutant forms of p53 are often highly expressed in tumors, but these mutations can produce complex effects. For instance, the R273H and R248Q mutations compromise DNA binding, while the R175D mutation completely abolishes p53's ability to induce cell cycle arrest and apoptosis^65^. Additionally, p53 is regulated by the ubiquitin-proteasome system, where MDM2 targets p53 for degradation as part of a negative feedback loop. However, certain p53 mutants may fail to activate MDM2, resulting in the loss of this regulatory mechanism^66^.

#### 4.1.2 Hyperactivity of anti-apoptotic pathways

Increased activity in anti-apoptotic signaling pathways is closely associated with apoptosis resistance. Mitochondrial outer membrane permeabilization and the release of cytochrome *c* into the cytoplasm are critical events in the initiation of intrinsic apoptosis. The release of cytochrome *c* is regulated by the Bcl-2 family proteins, which control mitochondrial membrane permeability. Once released, cytochrome *c* activates a cascade of caspases, leading to the execution of various cellular processes characteristic of the apoptotic program through protein hydrolysis. The Bcl-2 family consists of anti-apoptotic proteins, such as Bcl-2, Bcl-xL, Bcl-w, and Mcl-1, as well as pro-apoptotic effectors like Bax and Bak, and pro-apoptotic BH3-only proteins, including Bad, Bim, and Bid^67^. BH3-only proteins interact with anti-apoptotic Bcl-2 family members, releasing Bax and Bak to initiate apoptosis. The balance between these anti-apoptotic and pro-apoptotic proteins ultimately determines whether apoptosis will occur^68^.

In senescent cells, upregulation of anti-apoptotic Bcl-2 family proteins, particularly Bcl-2, Bcl-xL, and Bcl-w, is commonly observed. Evidence suggests that Bcl-2 overexpression promotes premature senescence when oxidative stress surpasses a critical threshold^69^. One study found that senescent cells were unable to downregulate Bcl-2, due to the prolonged activation of the transcription factor cAMP response element-binding protein, a positive regulator of Bcl-2, while cAMP response element-binding protein is able to be reduced in young cells^70^. Similarly, another anti-apoptotic protein, Bcl-xL, shows elevated transcript levels in senescent cells^71^. Likewise, under UV-induced apoptosis, senescent cells exhibit distinct regulation compared to young cells. Notably, instead of decreasing, the Bcl-xL protein of senescent cells increased, resulting in elevated resistance to apoptosis and ultimately a 10–20-fold reduction in apoptosis^72^. Additionally, senescent cells demonstrated upregulation of Bcl-w, and its overexpression similarly contributes to the promotion of senescence^73^^,^^74^. It has also been reported that the pro-apoptotic gene *BAX* is enriched with inhibitory histone marks in senescent cells^75^.

Parallelly, the overexpression of anti-apoptotic Bcl-2 family proteins is associated with poor prognosis and treatment resistance in various cancers^76^. Their roles in apoptosis resistance facilitate tumor growth and progression while also contributing to chemotherapy resistance^77^. For instance, Bcl-2 overexpression has been shown to accelerate c-Myc-driven lymphoma development in mice^78^. Moreover, Bcl-2 overexpression renders malignant cells resistant to anticancer drugs, through both p53-dependent and p53-independent mechanisms^79^. Bcl-xL is also highly expressed in multiple tumor types and plays a critical role in tumor progression^80^. Apoptosis resistance in tumors is further linked to restricted activation of the pro-apoptotic protein Bax, and overcoming these mechanisms to restore apoptosis remains a key strategy in combating apoptosis resistance in cancer^81^.

#### 4.1.3 Dysregulation of inflammatory signaling pathway

NF-*κ*B expression is upregulated in various tissues with age, and NF-*κ*B inhibition has been shown to delay the onset of aging-related diseases like diabetes and atherosclerosis^82^. Various DNA damage stimuli can activate NF-*κ*B signaling, which stimulates SASP production in senescence and regulates gene expression for apoptosis, cell cycle progression, and inflammation. This senescence-associated hyperactivation is partly mediated by the NF-*κ*B pathway and the subsequent activation of multiple secretory factors such as IL-1b, IL-6, and IL-8^83^. In addition, NF-*κ*B is responsible for apoptosis resistance in tumors due to modulation of inflammatory responses and oxidative stress, and its inhibition has been shown to induce apoptosis, inhibit angiogenesis, and delay sensitization of tumor growth^84^. In inflammation-induced carcinogenesis, NF-*κ*B signaling activates the transcription of multiple pro-tumorigenic cytokines and anti-apoptotic survival genes, such as Bcl-xL and the inhibitor of apoptosis (IAP). Subsequently, the BIR domain of the IAP binds to caspases and inhibits the execution phase of apoptosis^85^^,^^86^. NF-*κ*B pathway can also resist TNF-*α* and reactive oxygen species (ROS)-mediated apoptotic signaling by inhibiting JNK^87^.

#### 4.1.4 Other mechanisms of apoptosis resistance

Autophagy and apoptosis share signaling pathways and crosstalk protein regulation. Autophagy can block the induction of apoptosis, while activation of apoptosis-related caspase cascades shuts down the autophagy mechanism^88^^,^^89^. Heat shock proteins (HSPs) are a group of proteins that are produced by cells in response to stressful conditions, contributing to the proper folding, assembly, and functioning of proteins, and can prevent harmful protein aggregation. HSPs regulate apoptosis and generate resistance to apoptotic cell death in extensive ways, such as binding to Bax proteins and blocking their relocalization, binding to Bid and inhibiting its mitochondrial redistribution, as well as inhibiting apoptotic body formation^90^. HSP90 is an anti-apoptotic and pro-survival factor in SnC, and inhibitors of HSP90 have been identified as a new class of senolytic^33^. HSP expression is also significantly elevated in tumors, and inhibition of HSP has emerged as a novel therapeutic strategy in tumor therapy^91^. In addition, it is noticed that epigenetic regulation has also been shown to be strongly associated with apoptosis resistance in tumors and aging. Studies have shown that increased apoptosis resistance in cancer may be caused by DNA hypermethylation of some apoptosis-regulating genes, such as hypermethylation of the promoter, which will down-regulate the XAF1 protein, which in turn reduces the inhibitory effect of the anti-apoptotic XIAP protein^92^. Besides, histone modifications alter Bcl-2 and Bax gene expression in senescent fibroblasts, resulting in an apoptosis-resistant phenotype^75^.

### 4.2 The similar metabolic features of senescent cells and tumor cells

Senescence and cancer are two interrelated biological phenomena that share multiple metabolic characteristics, particularly in the way cells manage energy production and nutrient utilization^93^^,^^94^. Moreover, both senescent cells and tumor cells exhibit altered metabolic profiles that facilitate the survival and proliferation of abnormal cells^95^. These metabolic alterations include shifts toward glycolysis, altered lipid metabolism, mitochondrial dysfunction, and a dysregulated response to oxidative stress^96^. Recent studies have shown that these similar metabolic vulnerabilities can be targeted therapeutically, particularly using senolytic drugs, which are designed to selectively eliminate senescent cells or modulate their associated pathways. Therefore, understanding these metabolic alterations provides a basis for the development of therapeutic strategies targeting the common metabolic vulnerabilities of both conditions.

Glycolysis is a common metabolic feature of both senescent cells and tumor cells^97^^,^^98^. Tumor cells generate energy through aerobic glycolysis, known as the Warburg effect, a metabolic shift also widely adopted by senescent cells^99^^,^^100^. Glycolysis provides not only ATP but also pivotal metabolic intermediates for biosynthetic pathways required for cell growth, such as nucleotides, amino acids, and lipids^101^. This shift in metabolism is also observed in senescent cells, where enhanced glycolysis has been linked to the SASP and contributes to inflammation, tissue dysfunction, and the progression of age-related diseases, including cancer^102^. Both senescent cells and tumor cells rely on glycolytic intermediates for biosynthesis, making them metabolically vulnerable to interventions targeting glucose metabolism.

Senescent cells and tumor cells also display significant alterations in lipid metabolism. For instance, the discovery in Drosophila reveals that senescent glial cells promote lipid accumulation in non-senescent glial cells. Targeted intervention of AP1 activity in senescent glial cells can reduce senescence biomarkers, extend lifespan and health-span in *Drosophila*, and prevent lipid accumulation^103^. Lactate secreted by cancer-associated fibroblasts enhances lipid accumulation in lipid droplets within prostate tumor cells by increasing the expression of lipid metabolism-related genes, ultimately regulating the tumor's epigenetic pathways^104^. Tumor cells, particularly those with rapid proliferative activity, require increased lipid synthesis to build cellular membranes^105^. Lipid metabolism in tumor cells is often upregulated by enzymes such as fatty acid synthase and acetyl-CoA carboxylase^106^. Similarly, in senescence, lipid accumulation and altered lipid homeostasis have been linked to the development of age-related diseases. Senescent cells exhibit increased lipid droplet accumulation, which is associated with inflammation and a pro-tumorigenic environment^103^. The upregulation of lipid metabolism in both senescent cells and tumor cells represents a similar metabolic feature that can be targeted therapeutically.

Mitochondria play crucial roles in cellular energy production, and their dysfunction is a central feature of both senescence and cancer. In tumor cells, mitochondrial dysfunction often leads to increased production of ROS, which in turn contributes to genomic instability, a key driver of tumorigenesis^107^. In senescent cells, mitochondrial dysfunction leads to the accumulation of ROS, which damages cellular components, accelerates senescence, and promotes the SASP^2^. The connection between mitochondrial dysfunction, ROS, and cancer is well-documented, as ROS-induced DNA damage contributes to the mutation burden in tumors. Similarly, the accumulation of ROS in senescent cells accelerates cellular damage and promotes age-related diseases^108^.

Protein homeostasis, or proteostasis, is essential for maintaining cellular function. In both senescence and cancer, proteostasis is disrupted, leading to the accumulation of misfolded proteins. In cancer, this disruption is often linked to increased protein synthesis and a high demand for chaperone proteins to assist in protein folding. In KRAS inhibitor-resistant tumors, activation of both ERK and AKT restores IRE1*α* phosphorylation and stability, leading to proteostasis reprogramming^109^. Similarly, in senescence, the accumulation of damaged proteins and dysfunctional proteostasis contributes to cellular senescence and aging-related diseases. For example, gene dosage imbalance in senescent cells induces proteotoxic stress and activates major protein quality control mechanisms, while near-saturation of autophagy leads to disruption of proteostasis^110^. Autophagy, a cellular process that removes damaged proteins and organelles, is critical for maintaining proteostasis. In both senescence and cancer, autophagy is often impaired, leading to the accumulation of damaged cellular components^111^. Enhancing autophagy has been suggested as a potential therapeutic strategy for both senescence and cancer^112^.

### 4.3 The similar secretory phenotypes of senescent cells and tumor cells

Both tumor cells and senescent cells exhibit distinctive characteristics in their secretory phenotypes. Firstly, senescent cells typically display a feature known as the “SASP”, which refers to a group of active molecules secreted by senescent cells during the aging process, including proteins, interleukins, cytokines, chemokines, growth factors, and matrix metalloproteinases, etc^15^. These secretions can influence the surrounding cellular environment and have influential effects on tissue inflammation and regeneration. Tumor cells also often exhibit a similar secretory phenotype, secreting cytokines and growth factors such as VEGF, IL-6, and secreted transforming growth factor-beta (TGF-*β*), which contribute to tumor growth, metastasis, and immune evasion^113^. These secretions can induce alterations in the tumor microenvironment, promoting angiogenesis and the survival of tumor cells. In addition, modulating these secretory phenotypes can effectively inhibit tumor progression and improve senescence. In this section, we will describe the similar secretory phenotypes between cancer and senescent cells and summarize targeted small-molecule drugs based on corresponding mechanisms.

Firstly, senescent cells and tumor cells often secrete large amounts of cytokines such as IL-1*α*, IL-6, and IL-8, leading to a local inflammatory response. IL-6 is a pleiotropic cytokine that functions as both an autocrine and paracrine tumorigenic factor, with senescent cells serving as a significant source of this cytokine. Oncogene-induced cellular senescence (OIS) is a potent cancer-protective response capable of eliminating early-stage tumor cells. IL-6 is a central regulator of the inflammatory network-mediated OIS, and depletion of these specific interleukins, including IL-8, can lead to the collapse of the inflammatory network and prevent the entry and maintenance of senescence^114^. Moreover, IL6-and IL8-induced inflammation and senescence facilitate the acquisition of EMT and stem cell-like features, thus inducing breast tumor cells to develop an aggressive phenotype and increasing tumor aggressiveness^115^. Furthermore, senescent T cells can secrete the pro-inflammatory cytokines IL-6, IL-8, and TNF*α*, which can induce premature senescence of peripheral cells such as T cells through a paracrine mechanism, thus providing suppression of anti-tumor immunity^116^. In addition, IL-1*α* is a crucial regulator of the senescence-associated IL-6/IL-8 network. Mature IL-1*α* is generated during inflammasome activation and cellular senescence and subsequently involved in SASP^117^. The role of IL-1*α* in cancer remains controversial. It promotes tumorigenesis by promoting cell proliferation, activating angiogenesis to promote metastasis, as well as maintaining a tumor microenvironment conducive to tumor survival by regulating inflammatory factors. On the other hand, it is also involved in activating anti-tumor immune responses^118^.

Besides, senescent cells and tumor cells also secrete similar chemokines, such as CCL2, CCL5, and CXCL1. CCL2 is one of the key chemokines secreted by senescent cells *via* SASP, which affects neighboring cells through autocrine or paracrine effects, and it is significantly increased, especially in cancer-associated senescent cells. At an early stage of cancer development, oncogene-induced senescent hepatocytes secrete CCL2 and recruit CCR2^+^ myeloid cells, which mediate the clearance of senescent cells to prevent tumorigenesis, a phenomenon known as “senescence monitoring”^119^. Furthermore, CCL5 is able to synergize with CCL2 and other factors to activate the Hedgehog and TGF-*β* pathways, which promotes metastasis and spread of tumor cells^120^. On the contrary, senescent melanoma cells secrete CCL5, which will induce therapy-induced senescence and enhance tumor-infiltrating leukocytes' killing of tumor cells by promoting lymphocyte recruitment^121^. CXCL1 is also one of the chemokines secreted by senescent cells, which promotes tumor cell invasion and metastasis by activating the integrin *β*1/FAK/AKT signaling pathway^122^.

Moreover, senescent cells and tumor cells also secrete various growth factors. VEGF is one of the secretory phenotypes secreted by both senescent cells and tumor cells. Senescent cells secrete VEGF through SASP to promote angiogenesis, which provides more blood supply to tumor cells^123^. This mechanism plays a crucial role in tumor progression and metastasis. TGF-*β* is another important factor in SASP, and senescent cells regulate intercellular signaling by secreting TGF-*β* through SASP. TGF-*β* affects senescent cells both autocrinally and paracrinally, and together with VEGF, it can induce cellular senescence in the surrounding normal cells *in vitro* (called paracrine senescence)^124^. In addition, TGF-*β* both influences the fate of senescent cells themselves by promoting EMT and regulating immunosuppressive cells, and exacerbates tumor progression through pro-carcinogenic and immunosuppressive modalities in the tumor microenvironment^125^.

### 4.4 The epigenetic reprogramming of senescent cells and tumor cells

Although senescent cells and tumor cells have different ultimate fates—senescent cells exhibit proliferative arrest while tumor cells undergo uncontrolled proliferation—both undergo complex epigenetic reprogramming, leading to dysregulation of key genes. Senescent cells and tumor cells share similarities in their epigenetic alterations, primarily in gene expression regulation, chromatin structure remodeling, and epigenetic modifications such as DNA methylation and histone modifications^126^. These similar epigenetic changes may serve as important drivers of cellular dysfunction. Epigenetic alterations are fundamental to both senescence and cancer development, making them promising targets for therapeutic intervention. Epigenetics-based senolytic drugs, many of which are derived from antitumor drugs, have shown potential in selectively eliminating senescent cells and mitigating their harmful effects on tissue health. These dual functions highlight the potential of epigenetic therapies to address age-related diseases and cancer simultaneously, offering new avenues for enhancing healthspan and treating complex diseases associated with aging.

Senescent cells and tumor cells share similar mechanisms and features in epigenetic alterations, which have profound impacts on gene expression and cell fate. Firstly, changes in DNA methylation patterns are a key characteristic of both processes. In senescence, there is a global decrease in genomic DNA methylation levels, leading to genomic instability; meanwhile, abnormal hypermethylation occurs in promoter regions of certain genes, resulting in the silencing of tumor suppressor genes^127^^,^^128^. This heterogeneous methylation alteration is also widely observed in cancer and senescent cells. Secondly, abnormalities in histone modifications, such as dysregulation of histone acetylation and methylation, affect chromatin structure and gene expression. In senescent cells, decreased levels of Histone 3 lysine 9 trimethylation lead to the loss of heterochromatin and activation of transposons, increasing genomic instability^96^^,^^129^. In tumors, abnormal histone modifications can activate oncogenes or silence tumor suppressor genes, promoting tumor initiation and progression. In addition, changes in the expression of non-coding RNAs, such as microRNAs and long non-coding RNAs, play crucial roles in regulating gene expression and cellular functions. Abnormal expression of specific microRNAs can influence cell cycle progression, apoptosis, and senescence—processes essential in both aging and cancer development^130^^,^^131^. For example, the miR-17-92 cluster is overexpressed in various cancers and is believed to participate in the regulation of senescence pathways^132^. Moreover, there is significant interplay between metabolic reprogramming and epigenetic modifications. Metabolites such as acetyl-CoA, *S*-adenosylmethionine, and nicotinamide adenine dinucleotide serve as substrates or cofactors for epigenetic enzymes, directly affecting epigenetic regulation and influencing the development of senescence and cancer^133^^,^^134^. Recent studies have also highlighted the roles of chromatin remodeling complexes and telomere-associated epigenetic changes in these processes, indicating that the epigenetic landscape is central in both senescence and tumorigenesis^93^^,^^127^.

### 4.5 The immunity surveillance of senescent cells and tumor cells

Senescent cells and tumor cells share many similarities and intrinsic connections in immune regulation. For example, senescent cells can evade immune surveillance by secreting immunosuppressive factors that modulate the immune microenvironment, while tumor cells can achieve immune evasion through mechanisms such as upregulation of immune checkpoints^135^. Both senescent cells and tumor cells are capable of secreting pro-inflammatory factors that induce chronic inflammatory responses, thereby attracting immunosuppressive cells and facilitating immune escape. Furthermore, the accumulation of senescent cells leads to a decline in immune system function, further reducing the body's ability to eliminate both senescent cells and tumor cells^136^. These similarities offer novel insights for the discovery of senolytic drugs and anti-cancer therapies, highlighting the promising potential of strategies aimed at harnessing the immune system to clear senescent cells and treat tumors.

Programmed cell death protein 1 (PD-1) and its ligand programmed death-ligand 1 (PD-L1) are critical immune checkpoints in immunotherapy, and in recent years, blocking the PD-1/PD-L1 interaction has emerged as a powerful strategy in cancer immunotherapy^137^. Similar to tumor cells, senescent cells also exhibit overexpression of PD-1 and PD-L1 in certain contexts^17^. Furthermore, the use of anti-PD1 therapy has been shown to effectively alleviate the senescent phenotype and contribute to the treatment of type 1 diabetes^138^. CD47 is another key immunoregulatory protein that, by binding to signal regulatory protein alpha, inhibits macrophage phagocytosis and mediates the “don't eat me” signal^139^. The CD47–SIRP*α* interaction inhibits the phagocytic activity of macrophages, allowing tumor cells to escape immune surveillance and destruction. CD47 is overexpressed on the surface of many types of tumor cells, and studies have shown that senescent cells also evade immune surveillance by inhibiting macrophage recognition through the overexpression of CD47 on their surface, thereby facilitating immune escape^140^. Furthermore, senescent cells and tumor cells share similar secretory phenotypes to regulate immunity. Firstly, both tumor cells and senescent cells can induce chronic inflammation and achieve immune evasion by secreting pro-inflammatory cytokines such as IL-6, IL-10, and TNF-*α*, which suppress the activity of local immune cells^141^. Secondly, they can also secrete TGF-*β*, which inhibits T cell proliferation and promotes the differentiation of regulatory T cells, thereby enhancing immune suppression. Furthermore, other secretory phenotypes, such as VEGF and GM-CSF, can also contribute to immune system dysregulation. Additionally, senescent cells are capable of inducing tumorigenesis and tumor progression by modulating immune responses. Senescent cells can affect the changes of tumor immune cells by remodeling the TME, thus affecting the occurrence and development of tumor^142^. The abnormal proliferation signal produced by an oncogene mutation is called OIS. The effect of OIS on tumor cells is related to the balance between immune monitoring and immune escape. In the aging mouse model, CD4^+^ T cells were activated to eliminate tumor cells. Senescent HCC cells activate immune cells by secreting SASP factors, thereby mediating innate immunity and inhibiting tumor progression^143^. However, advanced immune regulation can promote tumor invasion and recruit immunosuppressive cells^144^.

In addition, the residual SASP in the immune system after aging can also damage the function of NK cells and promote tumorigenesis by recruiting immature myeloid cells^119^. The reduction of specific cytokines in TME can change the immunosuppressive characteristics of SASP, thus promoting the transition from immunosuppression to active immune surveillance^145^. The senescent tumor cells treated with chemotherapy drugs can be used as immune adjuvants to enhance the anti-tumor immune response. Chemotherapeutic drugs can activate NK cell-mediated immune surveillance in tumor models through NF-*κ*B-mediated SASP^146^, induce SASP to secrete cytokines, make tumor cells respond to PD-1 treatment, trigger the activation of CD8^+^ T cells, and enhance dendritic cell infiltration^147^. Chemotherapy-induced aging can also induce cancer immunosuppression. For example, some chemotherapeutic drugs promote chemoresistance by inhibiting the secretion of SASP factors induced by NK and T cells^145^. Senescent cells induce aging by recruiting immune cells, thereby affecting the occurrence and development of tumors^148^. Aging can remodel the immune system in TME by regulating SASP and show the characteristics of immune activation or immunosuppression. Aging cells induce aging by recruiting immune cells, thereby affecting the occurrence and development of tumors^148^. Aging can remodel the immune system in TME by regulating SASP and show the characteristics of immune activation or immunosuppression. In addition, senescent tumor cells exert M1 macrophage-mediated antitumor effect and M2 macrophage-related tumor-promoting activity through the epigenetic changes of macrophages mediated by SASP^149^.

## 5 Repurposing anti-tumor drugs as senolytic drugs based on meta-hallmarks

The current discovery of senolytic drugs relies on two primary approaches: a hypothesis-driven, bioinformatics-informed approach and high-throughput compound library screens. We found that most senolytic drugs based on these methods are repositioned from anti-tumor drugs, and their therapeutic mechanisms are closely aligned to the meta-hallmarks shared by senescent cells and tumor cells. Therefore, we classified existing senolytic drugs according to meta-hallmarks between senescent cells and tumor cells and discussed the potential molecular mechanisms in depth, providing a new clue for the development of senolytic drugs.

### 5.1 Senolytic drugs: From anti-tumor drugs that regulate apoptosis resistance

Given the similar apoptosis-resistant characteristics of senescent cells and tumor cells, there is potential for drug repurposing between antitumor treatments and senolytic therapies. For instance, reactivating normal p53 activity is a promising therapeutic strategy to slow both aging and tumor progression. Several small-molecule drugs have been developed to protect p53 from degradation by inhibiting its interaction with MDM2 or directly targeting MDM2, a negative regulator of p53^150^. For example, the Nutlin-3 series serves as a p53 activator and is widely used in antitumor research, among which Nutlin-3a is capable of preventing and reversing experimental pulmonary hypertension and may hold promise as a therapeutic agent for anti-senescence treatment for pulmonary hypertension^151^. RG-7112, an MDM2 inhibitor, has demonstrated antitumor activity and has already been in clinical trials in hematologic tumors. Recent research reported it can selectively induce apoptosis in senescent intervertebral disc cells, showcasing senolytic activity^152^. Another promising compound, BI01, can reduce senescent cells and promote muscle remodeling in aged mice by inhibiting p53–MDM2 binding^153^.

Furthermore, ubiquitin-specific peptidase 7 increases p53 levels by modulating MDM2 degradation. P5091 and P22077, which target ubiquitin-specific peptidase 7, have been shown to inhibit proliferation and induce apoptosis in colorectal cancer and neuroblastoma^154^^,^^155^. They are later found to be effective in suppressing senescence and mitigating the senescence-associated secretory phenotype in mice^156^. While many p53-targeting drugs are commonly used in cancer therapy, the novel mechanisms of the p53 pathway in aging could expand their application to age-related diseases. In addition to cell cycle regulation by the p53 network, recent studies have found that an extract from the natural plant sage Haenkenium reduces acute senescence induced by the chemotherapeutic drug adriamycin, whose senolytic mechanism stems from its disruption of the p16–CDK6 interaction^157^.

Another promising therapeutic approach for combating apoptosis resistance is inhibiting the Bcl-2 family of anti-apoptotic proteins. BH3 mimetics, such as ABT-737 and ABT-263 (navitoclax), can inhibit Bcl-2, Bcl-xL, and Bcl-w. Although initially developed for cancer treatment, these compounds have demonstrated senolytic activity, selectively eliminating senescent cells. Previous studies have identified that ABT-263 selectively eliminates senescent cells by inducing senescence and can rejuvenate senescent tissue stem cells^57^. ABT-737 treatment was also shown to reduce senescent microglia, improving cognition and brain inflammation in Alzheimer's disease model^158^. In addition, its treatment may help promote organ regeneration^159^. However, their hematological toxicity has limited their broader use^160^. Advances in protein degradation-targeted chimera (PROTAC) technology led to the development of PZ15227, a compound that reduces the platelet toxicity of navitoclax while preserving its ability to eliminate senescent cells. PZ15227 was shown to remove senescent cells and rejuvenate tissue stem and progenitor cells in naturally aging mice^161^. 753B is also a novel Bcl-xL/Bcl-2 PROTAC that avoids the platelet toxicity of ABT-263. It exhibits single-agent activity in AML, and can be synergistically treated with chemotherapy to overcome drug resistance^162^. Another ABT263-derived PROTAC drug, DT2216, is applied in the field of tumor therapy with much lower toxicity^163^. Additionally, A1331852 and A1155463, the second-generation Bcl-2 inhibitors, have induced apoptosis in senescent cells with greater target specificity and reduced hematotoxicity compared to navitoclax, positioning them as better candidates for clinical interventions^164^. A recent study identified three novel senolytic drugs, BRD-K20733377, BRD-K56819078, and BRD-K44839765, targeting Bcl-2 based on AI screening, which attenuated the degree of senescence in aged rats. Among them, BRD-K56819078 showed superior senescent cell selectivity and oral utilization to ABT-737 and is expected to be a more preferred anti-aging drug^165^. In addition, some natural products have apoptosis-related senolytic activities. For example, the curcumin analog, EF24, promotes the proteasomal degradation of the anti-apoptotic protein Bcl-2, which exerts an effective broad-spectrum anti-aging effect^35^. Cycloastrageno has been reported to play an inhibitory role in multiple tumors as well as ameliorate age-related physical dysfunction in animals recently. Its senolytic effects are associated with the inhibition of the Bcl-2 proteins and the PI3K/AKT/mTOR pathway, as well as the inhibition of the effects of SASP^166^.

Senescent tumor cells have been found to rely on the anti-apoptotic gene Mcl-1 for survival. Treatment with the Mcl-1 inhibitor S63845 effectively eliminated senescent tumor cells and metastases in mice, surpassing the efficacy of navitoclax. Other compounds, such as UM177 and AZD5991, have also shown senolytic activity in chemotherapy-induced senescent pancreatic tumor cells^167^. Besides, BTSA1 can directly activate pro-apoptotic proteins BAX, which not only overcomes apoptosis resistance in various solid tumors and hematologic cancers, but also is identified as a novel anti-aging drug for the treatment of pulmonary fibrosis^168^. There is significant crosstalk between the mitochondrial apoptotic pathway and cell cycle regulation. Inhibition of Bcl-xL has been found to activate p53, shifting cells from a senescent state to apoptosis, suggesting that wild-type p53 can be targeted to enhance cancer therapies that are based on Bcl-xL inhibition^169^. EE-84, derived from Aplysinopsins, is capable of inducing a senescent phenotype by inhibiting Bcl-2 and shows potential in the treatment of chronic myelogenous leukemia^170^.

**Figure 2:**
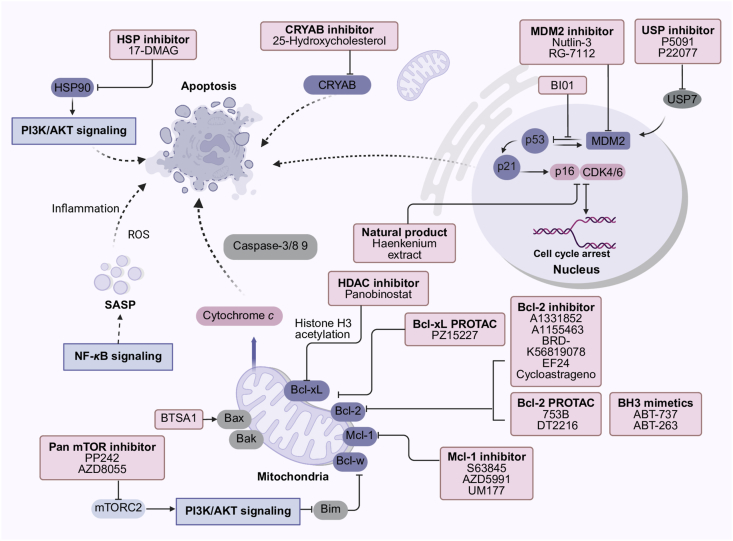
Senolytic drugs clear senescent cells by inhibiting apoptosis resistance. Senolytic drugs induce apoptosis of senescent cells through various pathways, such as regulating the expression level of apoptosis-related protein (Bcl-2, Bax, Bcl-xL, etc.); inhibiting the interaction between p53 and MDM2; promoting ROS generation; and suppressing PI3K/AKT signaling pathways.

In addition to targeted agents associated with the p53 network and apoptotic protein family, there are other mechanisms that can be utilized as targeting strategies for tumor and senescence therapies. Rapamycin is applied as an autophagy activator by targeting mTOR1 inhibition. It is widely used in tumor therapy and later is demonstrated to be effective in the inhibition of cellular senescence through mechanisms associated with restoration of autophagic fluxes^171^. The pan-mTOR inhibitors PP242 and AZD8055 have also been shown to be more effective than rapamycin in the treatment of senescence in sensitizing navitoclax-induced senescent apoptosis by increasing the expression of the proapoptotic protein Bim^172^. Meanwhile, the autophagy inhibitor chloroquine was able to direct cells undergoing BRAF senescence toward senolysis by modulating autophagy^173^. Inhibitors of HSP90 have been identified as a new class of senolytic, like 17-DMAG, which reduces the level of phosphorylated AKT and selectively induces apoptosis in senescent cells, prolonging the healthy lifespan of mice and delaying the onset of several age-related symptoms^33^. Another heat shock protein, CRYAB, is a senolytic target identified through extensive single-cell RNA sequencing efforts, and the known CRYAB inhibitor, 25HC, has been shown to have broad-spectrum senolytic activity through drug repositioning^174^. These drugs indicate the rational application of co-targeted apoptosis resistance mechanisms in senescent cells and tumor cells, providing directions for the retargeting application of more senolytic or antitumor drugs (Table 1, Fig. 2).

**Table 1:** The senolytic drugs, from anti-tumor drugs to regulate apoptosis and related mechanisms.

Active compd.	Mechanism as an anti-tumor drug	Mechanism as a senolytic/senomorphic drug	Application in senotherapeutics	Ref.
Nutlin-3	Serving as a p53 activator, and is widely used in antitumor research	Preventing and reversing experimental pulmonary hypertension through anti-senescence treatment	Pulmonary hypertension, skin, tumor	151,175
RG-7112	An MDM2 inhibitor has already been in clinical trials in hematologic tumors	Selectively inducing apoptosis in senescent intervertebral disc cells, showcasing senolytic activity	Vascular calcification, IVD, tumor	44,152
BI01	Increasing the abundance of both p53 and MDM2 and markers of apoptosis in osteosarcoma cells	Reducing senescent cells and promoting muscle remodeling in aged mice by inhibiting p53–MDM2 binding	Skeletal muscle, tumor	153
P5091, P22077	Targeting ubiquitin-specific peptidase 7 has been shown to inhibit proliferation and induce apoptosis in colorectal cancer and neuroblastoma	Suppressing senescence and mitigating the SASP in mice	Osteoporosis, cardiac hypertrophy, tumor	176,177
Natural plant sage Haenkenium	Reducing acute senescence induced by the chemotherapeutic drug adriamycin	Reducing acutely induced senescence by disrupting the p16–CDK6 interaction	Promote longevity	157
ABT-737, ABT-263 (navitoclax)	BH3 mimetics, initially developed for cancer treatment by inhibiting the Bcl-2 family of anti-apoptotic proteins	Selectively eliminating senescent cells by inhibiting the Bcl-2 family of anti-apoptotic proteins	Alzheimer's disease, atherosclerosis, and tumors	158,178
PZ15227	Reducing the platelet toxicity of navitoclax	Removing senescent cells and rejuvenating tissue	Rejuvenate tissue stem and progenitor cells	161
753B	A novel Bcl-xL/Bcl-2 protein degradation-targeted chimera (PROTAC) that avoids the platelet toxicity of ABT-263. It exhibits single-agent activity in AML	Eliminating leukemia cells and enhancing the efficacy of chemotherapy by targeting senescent cells	Tumor	162
DT2216	ABT263-derived PROTAC drug is applied in the field of tumor therapy with much lower toxicity	It has the potential to be developed as an anti-aging drug	Tumor	161
A1331852, A1155463	Reducing hematotoxicity compared to navitoclax, positioning them as better candidates	As second-generation Bcl-2 inhibitors, they have induced apoptosis in senescent cells with greater target specificity	Tumor	164
BRD-K20733377, BRD-K56819078 and BRD-K44839765	There are no tumor-related studies.	Targeting Bcl-2 based on AI screening attenuated the degree of senescence in aged rats.	Kidney aging	165
The curcumin analog, EF24	As an anti-tumor compound, to induce apoptosis, inhibiting proliferation and metastasis in various cancers.	Promoting the proteasomal degradation of the anti-apoptotic protein Bcl-2, which exerts an effective broad-spectrum anti-aging effect	Pulmonary fibrosis, tumor	35,179
Oridonin	Having robust anti-inflammatory activity and is capable of exerting anti-tumor effects by modulating cellular pyroptosis	Inducing apoptosis by activating the reactive oxygen species (ROS)–p38 signaling axis in senescent cells	Tumor	180
Cycloastrageno	Playing an inhibitory role in multiple tumors as well as ameliorating age-related physical dysfunction in animals	Its senolytic effects are associated with the inhibition of the Bcl-2 proteins and the PI3K/AKT/mTOR pathway, as well as the inhibition of the effects of SASP	Restore physical function, tumor	166
EE-84	Showing potential in the treatment of chronic myelogenous leukemia	Inducing a senescent phenotype by inhibiting Bcl-2	Tumor	170
S63845, UM177 and AZD5991	Effectively eliminated senescent tumor cells and metastases in mice, surpassing the efficacy of navitoclax	Having shown senolytic activity in chemotherapy-induced senescent pancreatic tumor cells	Tumor	167
BTSA1	Directly activating pro-apoptotic proteins BAX, which overcomes apoptosis resistance in various solid tumors and hematologic cancers	Having been identified as a novel anti-aging drug for the treatment of pulmonary fibrosis by activating BAX	Pulmonary fibrosis, tumor	168
PP242 and AZD8055	Inhibiting the proliferation of various tumor cells by suppressing protein synthesis	Sensitizing navitoclax-induced senescent apoptosis by increasing the expression of the proapoptotic protein Bim	Tumor	172
Chloroquine	As the most common drugs used to relieve acute and chronic inflammatory diseases by regulating autophagy, apoptosis, and necrosis	Directing cells undergoing BRAF senescence toward senolysis by modulating autophagy	Skin	173
17-DMAG	Inducing apoptosis of acute lymphoblastic leukemia cells by disrupting the autophagy flux	Reducing the level of phosphorylated AKT selectively induces apoptosis in senescent cells	Acute kidney injury in diabetes, tumor	33,181
25HC	There are no tumor-related studies.	Having been shown to have broad-spectrum senolytic activity through drug repositioning	Skeletal muscle	174

### 5.2 Senolytic drugs: From anti-tumor drugs that regulate metabolism

Tumor cells and senescent cells exhibit significant metabolic similarities, particularly in their dependency on glucose metabolism and alterations in energy production. With an increasing understanding of metabolic reprogramming, some antitumor drugs based on the regulation of tumor metabolism were repurposed as senolytic drugs.

Senolytic drugs such as quercetin and dasatinib modulate glycolysis by inhibiting key enzymes involved in this pathway, thus selectively eliminating senescent cells and interfering with the metabolic processes of tumor cells. Quercetin is a natural flavonoid compound that can inhibit tumor cell proliferation by reducing the activity of glycolytic enzymes. In recent years, it has been found to selectively eliminate accumulated senescent cells in the body and improve age-related diseases^182^. Dasatinib is a second-generation tyrosine kinase inhibitor that targets the Bcr-Abl tyrosine kinase and is primarily used in the treatment of various solid tumors and hematologic malignancies^183^. Recent research indicated that dasatinib inhibits crucial signaling pathways in senescent cells (such as the SRC family kinases), reducing the support for glycolysis, and inducing cell death^184^. 2-Deoxy-d-glucose, an effective glycolysis inhibitor, eliminates senescent cells by inducing an intrinsic apoptotic pathway; moreover, 2-deoxy-d-glucose also sensitizes tumor cells to chemo/radiation treatment^185^. Aminooxyacetic acid (AOAA) is known as an inhibitor of aspartate aminotransferase, which is a rate-limiting enzyme of the malate–aspartate shuttle. It's reported that AOAA can selectively inhibit the growth of breast adenocarcinoma in athymic mice and suppress the proliferation of breast tumor cells. Further study demonstrated that the treatment of AOAA decreased the level of senescent C6 glioma cells^38^.

Antitumor drugs regulating redox were also exploited as senolytic drugs to reduce ROS levels or enhance antioxidant systems, selectively eliminating senescent cells and restoring metabolic homeostasis. For example, quercetin and resveratrol are senolytic drugs that enhance the activity of antioxidant enzymes, thereby decreasing ROS levels and reducing the survival of senescent cells^182^^,^^186^. Flavonoid 4,4′-dimethoxychalcone is a flavonoid small-molecule compound that inhibits tumor cell proliferation by activating Keap1/Nrf2/HMOX1 pathway to induce ferroptosis^187^. Recently, it has been found to induce ferroptosis in senescent cells by promoting ferritinophagy, leading to an increase in the labile iron pool and subsequent cell death^188^. MitoQ, which specifically targets mitochondria, is being explored for its potential to reduce ROS production and alleviate both senescence and cancer-related pathologies^189^^,^^190^. Gingerenone A, extracted from ginger, not only inhibits tumor progression by inducing ferroptosis in colorectal cancer, but also inhibits oxidative stress-induced proliferation and senescence of breast tumor cells^191^^,^^192^. Oridonin from traditional Chinese medicine has robust anti-inflammatory activity and is capable of exerting anti-tumor effects by modulating cellular pyroptosis^193^. Recently, it has been identified as a novel senolytic drug, inducing apoptosis by activating the ROS–p38 signaling axis in senescent cells^180^.

Both senescent cells and tumor cells often manifest mitochondrial dysfunction or metabolic reprogramming, and senolytic drugs that restore mitochondrial function, such as metformin and dichloroacetate, have been shown to regulate mitochondrial metabolic pathways and combat both aging and cancer development^194^. Metformin, a pervasively used antidiabetic drug, enhances mitochondrial oxidative phosphorylation by activating AMP-activated protein kinase. This shift from glycolysis to oxidative phosphorylation reduces tumor cell proliferation and sensitizes them to therapy^195^. Dichloroacetate promotes the entry of pyruvate into the mitochondria by inhibiting pyruvate dehydrogenase kinase, enhancing oxidative metabolism, and reducing lactate production, which is beneficial in both senescence and cancer contexts^196^.

The triterpenoid complex from *Ganoderma lucidum* can synergize with chemotherapeutic agents to treat tumors by eliminating senescent cells *via* caspase-dependent and mitochondrial pathway-mediated apoptosis, among other mechanisms^197^. GL-V9 is a newly synthesized flavonoid derived from wogonin, which has demonstrated anti-tumor effects in various tumor cells. It's reported that GL-V9 can kill senescent cells, including chemotherapy- and replication-induced senescent cells, by alkalizing lysosomes and increasing the abundance of mitochondria as well as ROS^198^. Tamoxifen is known as a mitochondria-targeted anticancer agent. Further research verified that tamoxifen can selectively eliminate senescent cells *in vitro* and *in vivo* by suppressing OXPHOS and decreasing mitochondrial membrane potential in senescent cells, and therefore resulting in the loss of mitochondrial integrity and cell death^199^.

**Figure 3:**
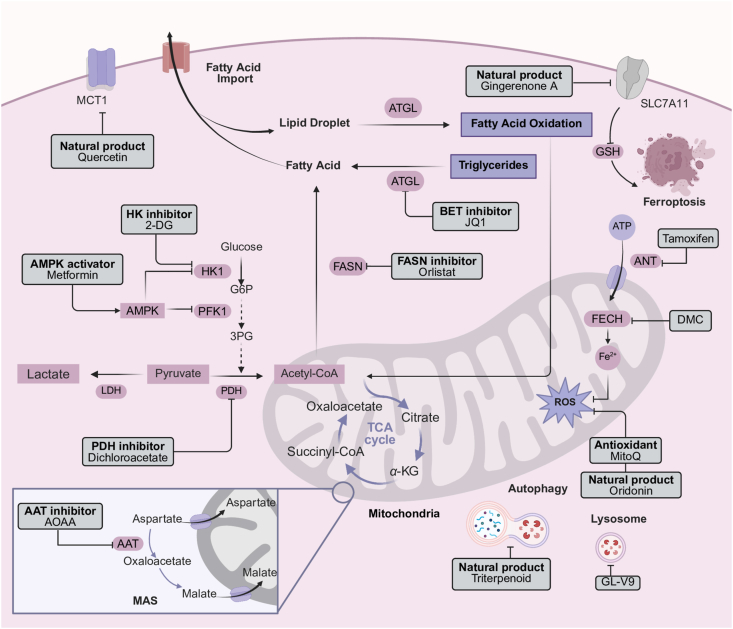
Senolytic drugs eliminate senescent cells by regulating metabolism. Senescent cells display various metabolic changes such as glycolysis, the Krebs' cycle, lipid metabolism, and ROS production, and senolytic drugs can eliminate senescent cells mainly by regulating the expression levels of these metabolism-related enzymes.

Additionally, lipid-regulating drugs have been shown to support the role of lipid metabolism in both cancer and senescence. For example, BRD4 inhibitor JQ1 can inhibit the proliferation of triple-negative breast cancer cells by adipose triglyceride lipase mediating lipolysis, and also can prevent the aging of LPS-induced senescent macrophages and lipid accumulation by reducing the expression of SASP in both autocrine and paracrine senescence^200^^,^^201^. Targeting lipid biosynthesis with fatty acid synthase inhibitors, such as orlistat, has shown promise in reducing cancer cell proliferation and inducing apoptosis. These compounds can disrupt the lipid metabolic support provided by senescent cells, thereby limiting their pro-tumorigenic effects^202^. Autophagy-inducing drugs, such as spermidine, have shown promise in preclinical studies for inhibiting cancer progression and extending lifespan^203^. Given the numerous similarities in metabolic regulation between senescent cells and tumor cells, as well as the repurposing of drugs targeting tumor metabolism as senolytic drugs, this provides important insights for the future development of senolytic drugs based on the regulation of tumor metabolism (Table 2, Fig. 3).

**Table 2:** The mechanisms of drugs to treat tumors and clear senescent cells by regulating metabolism.

The active compd.	Mechanism as an anti-tumor drug	Mechanism as a senolytic/senomorphic drug	Application in senotherapeutics	Ref.
Quercetin	Inhibiting tumor cell proliferation by reducing the activity of glycolytic enzymes	Promoting apoptosis of senescent cells by inhibiting the anti-apoptotic signal of BCL-2 family proteins in senescent cells	Pulmonary fibrosis, diabetes, Alzheimer's disease, intervertebral disc (IVD), osteoporosis	45,46
Dasatinib	As a second-generation tyrosine kinase inhibitor primarily used in the treatment of various solid tumors and hematologic malignancies	Inhibiting crucial signaling pathways in senescent cells (such as the SRC family kinases), reducing the support for glycolysis	Pulmonary fibrosis, diabetes, Alzheimer's disease, IVD, osteoporosis	45,46
2-Deoxy-d-glucose	As an effective glycolysis inhibitor, it sensitizes tumor cells to chemo/radiation treatment	Eliminating senescent cells by inducing an intrinsic apoptotic pathway	Osteoporosis, tumor	185
Aminooxyacetic acid (AOAA)	Selectively inhibiting the growth of breast adenocarcinoma in athymic mice and suppressing the proliferation of breast tumor cells	Decreasing the level of senescent C6 glioma cells	Tumor	38
Resveratrol	Tumor inhibition through multiple mechanisms, such as PI3K/AKT, ROS	Decreasing ROS levels and reducing the survival of senescent cells	Neurodegenerative diseases, cardiovascular diseases, and tumors	204
Flavonoid 4,4′-dimethoxychalcone	Inhibiting tumor cell proliferation by activating Keap1/Nrf2/HMOX1 pathway to induce ferroptosis	Inducing ferroptosis in senescent cells by promoting ferritinophagy, leading to an increase in the labile iron pool and subsequent cell death	Liver diseases, tumor	188
MitoQ	Targeting mitochondria and reducing ROS production	Reduce ROS production	Skin, pulmonary fibrosis, tumor	189,190,205
Gingerenone A	Inhibiting tumor progression by inducing ferroptosis in colorectal cancer	Inhibiting oxidative stress-induced proliferation and senescence of breast tumor cells	Tumor	191,192
GL-V9	Having demonstrated the anti-tumor effects in various tumor cells	Killing senescent cells, including chemotherapy- and replication-induced senescent cells, by alkalizing lysosomes and increasing the abundance of mitochondria and ROS	Liver fibrosis, tumor	198,206
Tamoxifen	By competitively blocking the estrogen receptor, the intracellular estrogen level is reduced, thus inhibiting the growth of tumor cells	Selectively eliminating senescent cells by suppressing OXPHOS and decreasing mitochondrial membrane potential	Diabetes, tumor	199
Metformin	From glycolysis to oxidative phosphorylation reduces tumor cell proliferation and sensitizes them to therapy	Enhancing mitochondrial oxidative phosphorylation by activating AMPK	Diabetes, tumor	195
Dichloroacetate	Promoting the entry of pyruvate into the mitochondria, enhancing oxidative metabolism and reducing lactate production	Promoting the entry of pyruvate into the mitochondria, enhancing oxidative metabolism and reducing lactate production	Tumor	196
The triterpenoid complex from *Ganoderma lucidum*	Synergizing with chemotherapeutic agents to treat tumors	Eliminating senescent cells *via* caspase-dependent and mitochondrial pathway-mediated apoptosis, among other mechanisms	Tumor	197
JQ1	Inhibiting the proliferation of triple-negative breast carcinoma cells by adipose triglyceride lipase (ATGL) mediating lipolysis	Preventing the aging of LPS-induced senescent macrophages and lipid accumulation	Atherosclerosis, tumor	200,201
Orlistat	Showing promise in reducing cancer cell proliferation and inducing apoptosis	Disrupting the lipid metabolic support provided by senescent cells	Tumor	202
Spermidine	Inhibiting cancer progression	Extending lifespan	Heart disease, neurological disease, tumor	203,207

### 5.3 Senolytic drugs: From anti-tumor drugs that regulate secretory phenotypes

Targeting the secretory phenotypes of tumor cells has achieved great success in inhibiting tumor proliferation. Similarly, modulating the SASP is also a potential strategy for improving aging. However, it is worth noting that there are several anti-cellular senescence strategies, including not only senolytic drugs, which eliminate senescent cells, but also senomorphics, which prevent the harmful extracellular effects of senescent cells, and the drugs used for regulating SASP to improve senescence are referred to as senomorphics^208^.

The mTOR pathway coordinates cell growth and metabolism in response to nutrients and promotes the production of secretory phenotypes, such as NF-*κ*B and IL-1*α*, by increasing the translation of a subpopulation of mRNAs and regulating autophagy and immune function^209^. Targeting mTOR through small molecules such as rapamycin, BAY 11-7082, and RAD001 can effectively improve both tumor suppression and aging-related processes. Rapamycin, an mTOR inhibitor, has been approved by the FDA for the treatment of lymphangioleiomyomatosis. In addition, rapamycin was discovered to extend lifespan in diverse species, including mice. Further studies have shown that rapamycin can reduce the levels of IL6 and other cytokine mRNAs by inhibiting mTOR and selectively suppresses the translation of the membrane-bound cytokine IL1A. This, in turn, decreases NF-*κ*B transcriptional activity, ultimately inhibiting the proliferation of tumor cells by improving the SASP of senescent cells^210^. Another mTORC1 inhibitor, compound 3, obtained by virtual screening, showed toxicity to tumor cells and anti-SASP activity^211^. In addition, acting as an ATM inhibitor, KU-60019 was applied in glioblastoma and later identified as senomorphic for its capability of restoring the function of the lysosomal/autophagic system and mitochondrial activity^212^.

The JAK/STAT signaling pathway also plays an important role in the regulation of inflammation and proliferation. The JAK/STAT pathway is highly upregulated in senescent cells and tumor cells compared to normal cells^213^^,^^214^. Ruxolitinib, a JAK inhibitor, is mainly used for the treatment of myelofibrosis and has been studied in many hematologic tumors. Recent studies have found that ruxolitinib pharmacologically inhibits SASP production and attenuates senescence^215^.

In addition, direct inhibition of inflammatory factors can alleviate the pro-carcinogenic effects in the tumor microenvironment and participate in aging-related diseases by ameliorating inflammation. Many neutralizing antibodies against key SASP components or their receptors, including IL-6, IL-8, IL-1, and TNF, also display senomorphic features. For instance, tocilizumab is a biologic agent used to reduce inflammatory responses by blocking the IL-6 signaling pathway, alleviating age-related chronic inflammation. It is primarily used to treat rheumatoid arthritis and other autoimmune diseases, and also demonstrates potential applications in the treatment of hematologic cancers and breast cancer by preventing IL-6's exogenous harmful effects (NCT05846789). Siltuximab is also a monoclonal antibody that directly targets IL-6 and has been used to treat Castleman's disease and has been studied in various tumors such as multiple myeloma, lymphoma, and leukemia (NCT05316116). Besides, simvastatin can affect inflammation and oxidative stress by inhibiting IL-6 and IL-8, which have shown anti-aging effects in preclinical models of senescence and demonstrated antitumor effects by impacting proliferation and migration^216^^,^^217^.

Reorafenib, a targeted drug for the treatment of advanced cancer, has recently been found to slow down aging and improve the age-related lung disease emphysema by repositioning drug screening. This effect is mainly through the effector pathways AKT/mTOR, ERK/RSK, and JAK/STAT3, which ultimately improve the secretion of IL-6 and IL-8, etc.^218^.

Anakinra is an antagonist targeting IL-1, which is commonly applied in inflammatory diseases and controlling aging-related diseases driven by inflammation. Currently, several clinical trials are exploring the potential role of Anakinra in cancer treatment. In addition, among the drugs targeting IL-1, Xilonix, primarily used in cancer, has shown potential in the treatment of diseases associated with chronic inflammation, which is related to a variety of aging diseases^219^. Infliximab and etanercept reduce inflammation and immune response by targeting TNF, which is mainly applied in autoimmune diseases, while the relationship with tumors is not clear. Rutin blocks the development of the SASP by interfering with the interactions between ATM and TRAF6, as well as between ATM and HIF1*α* in senescent cells^220^. Other targeted inhibitors of SASP components are all mainly applied in the field of oncology, such as bevacizumab (anti-VEGF), galunisertib (TGF-*β* inhibitor), carlumab (CCL2 inhibitor), leronlimab (anti-CCL5), SX-682 (CXCL1 inhibitor), etc. The application of these drugs in seno-therapeutic strategy remains to be explored^15^.

**Figure 4:**
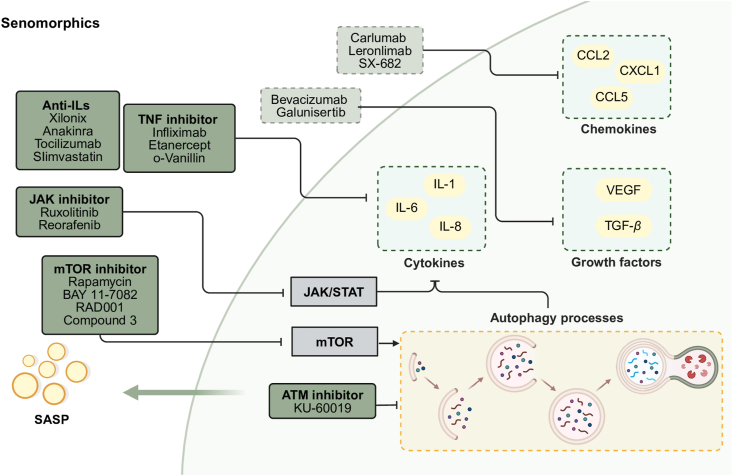
The primary senomorphics that regulate SASPs of senescent cells. Senescent cells can secrete SASPs, including chemokines, cytokines, and growth factors, and senomorphics can inhibit these SASPs by directly neutralizing proinflammatory factors or regulating JAK/STAT, mTOR signaling pathways, but not eliminate senescent cells.

Besides, *o*-vanillin, a natural anti-inflammatory agent, was effective in attenuating the pro-tumorigenic phenotype of microglia, and was also effective in reducing the SASP of Intervertebral disc cells^152^^,^^221^. *N*-*trans*-Feruloyltyramine, extracted from *Allium hookeri*, effectively inhibited SASP hallmarks and showed cytotoxicity against hepatocellular carcinoma (Table 3, Fig. 4)^222^.

**Table 3:** The mechanisms of drugs that regulate secretory phenotypes of senescent cells and tumor cells.

The active compd.	Mechanism as an anti-tumor drug	Mechanism as a senolytic/senomorphic drug	Application in senotherapeutics	Ref.
Rapamycin	Having been approved by the FDA for the treatment of lymphangioleiomyomatosis.	Extending lifespan to improve SASP by reducing the levels of IL6 and other cytokine mRNAs	Skeletal muscle, skin, ischemic diseases, extended life span, tumor	210
Compound 3	Having been obtained by virtual screening, showed toxicity to tumor cells	Inhibiting SASP activity by inhibiting mTOR	Tumor	211
KU-60019	Having been used to be applied in glioblastoma	Having been identified as senomorphic for its capability of restoring the function of the lysosomal/autophagic system and mitochondrial activity	Skin, tumor	212
Ruxolitinib	A JAK inhibitor, which is mainly used for the treatment of myelofibrosis and has been studied in many hematologic tumors	Pharmacologically inhibiting SASP production and attenuating senescence	Osteoporosis, age-related frailty,	215,223
Tocilizumab	It is used to treat hematologic cancers and breast cancer by preventing IL-6's exogenous harmful effects	Reducing inflammatory responses by blocking the IL-6 signaling pathway, alleviating age-related chronic inflammation	Hutchinson-Gilford progeria syndrome, tumor	224
Simvastatin	Having been demonstrated to have antitumor effects by impacting proliferation and migration	Affecting inflammation and oxidative stress by inhibiting IL-6 and IL-8, which has shown anti-aging effects in preclinical models of senescence	Skin, multiple sclerosis, tumor	217,225
Reorafenib	A targeted drug for the treatment of advanced cancer	Improving the age-related lung disease emphysema by regulating AKT/mTOR, ERK/RSK, and JAK/STAT3, which ultimately improves the secretion of IL-6 and IL-8, etc.	Pulmonary emphysema, tumor	218
Anakinra	Several clinical trials are exploring the potential role of Anakinra in cancer treatment.	As an antagonist targeting IL-1, which is commonly applied in inflammatory diseases and controlling aging-related diseases driven by inflammation	Vascular disease, tumor	226
Xilonix	Having demonstrated the therapeutic potential in non-small cell lung cancer	Having shown potential in the treatment of chronic inflammation, which is related to a variety of aging diseases	Chronic inflammatory diseases, tumors	219
Infliximab, etanercept	While the relationship with the tumor is not clear	Reducing inflammation and immune response by targeting TNF, which is mainly applied in autoimmune diseases	Osteoarthritis, tumor	227
Rutin	Inducing apoptosis of various tumor cells by regulating ROS and cytokines	Blocking the development of the SASP by interfering with the interactions between ATM and TRAF6, as well as between ATM and HIF1*α* in senescent cells	Atherosclerosis, tumor	220,228
*o*-Vanillin, extracted	A natural anti-inflammatory agent was effective in attenuating the pro-tumorigenic phenotype of microglia,	Having been demonstrated potential in reducing the SASP	IVD, tumor	152,221
*N*-*trans*-Feruloyltyramine	Showing cytotoxic against hepatocellular carcinoma	Effectively inhibiting SASP hallmarks	Neurological disease, tumor	222

### 5.4 Senolytic drugs: From anti-tumor drugs that regulate epigenetics

Epigenetics-based senolytic drugs have become a hotspot in anti-aging and anticancer research. By targeting and eliminating senescent cells, it's possible to alleviate their detrimental effects on the tissue microenvironment, thereby delaying the progression of aging-related diseases. Recent studies focus on developing senolytic drugs that modulate epigenetic regulatory factors.

HDAC inhibitors have been extensively studied for their roles in cancer therapy due to their ability to alter chromatin structure and gene expression, leading to cell cycle arrest and apoptosis in tumor cells ^229^. However, their role as senolytic drugs is complex. In some contexts, HDAC inhibitors may actually induce senescence rather than eliminate senescent cells. The over-activity of HDAC can result in chromatin packing and inhibit gene expression^230^. In senescent cells, increased HDAC activity may maintain the senescence phenotype. Panobinostat has been shown to exhibit antitumor activity in previous studies by inhibiting histone deacetylation to induce proliferation arrest and apoptosis of tumor cells^231^. As a senolytic, panobinostat can selectively induce chemotherapy-induced senescent tumor cell death by decreasing Bcl-xL expression and increasing histone H3 acetylation, thus significantly reducing the residual senescent cells after chemotherapy. It showed a higher clearance rate than repeated chemotherapy^22^. Fisetin is a natural flavonoid compound with strong antioxidant and anti-inflammatory properties. It inhibits the proliferation and metastasis of tumor cells by inhibiting HDACs and regulating cell cycle and apoptosis pathways^34^. As a potential senolytic agent, fisetin has shown stronger effects than quercetin in multiple *in vitro* and *in vivo* models. It can selectively remove senescent cells and reduce the release of SASP factors, thereby alleviating inflammation and tissue dysfunction associated with aging^164^.

Compounds that activate the sirtuin family HDACs have been investigated as potential anti-aging and anti-tumor drugs. These compounds not only extend the life span of many organisms, but also prevent and treat age-related diseases, including cancer, by clearing senescent cells^128^. Resveratrol, as an activator of Sirtuin 1 and 2, exerts potent antitumor effects by modulating epigenetic marks, reducing DNA methylation, influencing mitochondrial dynamics, and regulating cell metabolism, apoptosis, and autophagy through the SIRT1–p53 pathway^232^. Additionally, resveratrol has been shown to delay the aging process in adipose-derived stem cells by reducing cellular senescence, further contributing to its potential as a senolytic agent^233^. In addition, SRT1720 and SRT2104 have shown anticancer properties as activators of Sirtuin-1. SRT1720 contributes to the clearance of senescent cells by reducing oxidative stress and inflammation, which may inhibit tumor growth and metastasis^234^^,^^235^. SRT2104 may prevent tumor-induced muscle and bone wasting by protecting tissue integrity^236^. Recent studies have found that SRT2104 enhances tumor therapeutic efficacy by inhibiting small extracellular vesicles secreted by senescent cells through activation of SIRT1, reducing its promoting effect on drug resistance of tumor cells^237^.

Bromodomain and extra-terminal (BET) inhibitors refer to compounds targeting bromodomain and BET proteins, which are critical for recognizing acetylated histones and maintaining gene transcription^238^. JQ1, a BET inhibitor, blocks the binding of BET proteins to acetylated histones, thereby inhibiting gene expression and exhibiting strong antitumor effects. Interestingly, these antitumor properties led to the discovery that BET inhibitors may also act as senolytic drugs by reducing the expression of pro-survival genes in senescent cells, promoting their clearance^239^. For example, a degrader of the BET family proteins (BETd), identified through high-throughput screening and bio-functional analysis, has been shown to attenuate non-homologous end joining and upregulate the expression of autophagic genes. This mechanism promotes senolysis in senescent hepatic stellate cells^37^. ARV-825, a BRD4 PROTAC degrader, can effectively suppress the proliferation and induce apoptosis of T-cell acute lymphoblastic leukemia cells by perturbing the H3K27Ac-Myc pathway and reducing c-Myc protein levels. ARV-825 can also selectively kill senescent liver tumor cells, which were induced by the combination of CDK4/6 inhibitor and XPO1 inhibitor^240^.

Recent studies have identified epigenetic targets, such as lysine-specific demethylase 1 and enhancer of zeste homolog 2 (EZH2), which play essential roles in tumor progression by maintaining the silenced state of tumor suppressor genes. GSK343 is a selective EZH2 inhibitor that reduces H3K27me3 levels, reactivating silenced tumor suppressor genes, and inducing apoptosis or autophagy in tumor cells, thereby exhibiting strong antitumor effects^241^. Another compound, 3-deazaneplanocin A (DZNEP), is an EZH2 degrading agent that reduces EZH2 protein levels, leading to a decrease in H3K27me3 and reactivation of tumor suppressor gene expression, ultimately inducing apoptosis in tumor cells^242^. Interestingly, these antitumor properties of EZH2 inhibitors led to their identification as potential senolytic drugs. GSK343 and 3-deazaneplanocin A not only target tumor cells but also act on senescent cells by reversing senescence-associated epigenetic changes, reducing EZH2 levels, and promoting the clearance of senescent cells through apoptosis^243^^,^^244^.

### 5.5 Senolytic drugs: from anti-tumor drugs that regulate immune surveillance

In recent years, the regulation of immune therapy for tumors has achieved significant success, and these immunotherapies have also been extensively developed for the clearance of senescent cells. For example, immunomodulation against PD-1/PD-L1 has made significant progress in cancer therapy. Meanwhile, studies have shown that senescent cells also exhibit heterogeneous expression of PD-L1, and the addition of PD-1 antibodies can facilitate the clearance of PD-L1^+^ senescent cells in an activated CD8^+^ T cell-dependent manner^17^.

CD47 is a highly promising therapeutic target in cancer treatment. It can effectively eliminate tumor cells by restoring the phagocytic function of the immune system. Currently, several CD47-targeting drugs have been approved for phase III clinical trials for the treatment of various cancers^245^. In terms of senescent cells, the increased expression of CD47 enhances their resistance to macrophage-mediated phagocytosis and clearance, while also impairing the ability of macrophages to remove bystander apoptotic cells, and the CD47 antagonists eliminate senescent cells and maintain tissue homeostasis by inhibiting the SIRP*α*–CD47–SHP-1 axis^140^. Another study showed that the senescence inducer CDC7 inhibitor and the CD47 inhibitor AZD8055 co-loaded into the nanocarrier system were able to be sequentially released. Thus, the CDC7 inhibitor is released first to induce senescence in hepatocellular carcinoma cells, followed by the release of the CD47 inhibitor mediating an immune response to eliminate the senescent cells^246^. This also provides a therapeutic strategy for the use of senolytic Drugs after artificial induction of senescence.

Furthermore, since tumor cells and senescent cells both overexpress certain antigens on their membrane surfaces, antibody–drug conjugates (ADCs) targeting these antigens have been developed as senolytic drugs. For example, *β*2-microglobulin is a protein highly expressed in both senescent cells and tumor cells. Thus, targeting *β*2-microglobulin, an ADC linked to doxorubicin *via* a cleavage-enzymatic ligand, was designed to eliminate 35% of senescent cells in tumor cell lines ^247^. Apolipoprotein D antibodies are frequently utilized in the design of anti-tumor ADCs and can also be coupled with pyrrolo benzodiazepine drug PBD in the elderly mouse model, selectively eliminating the senescent skin fibroblasts *in vivo* and *in vitro*, and increasing the mechanical properties of the skin^248^.

**Figure 5:**
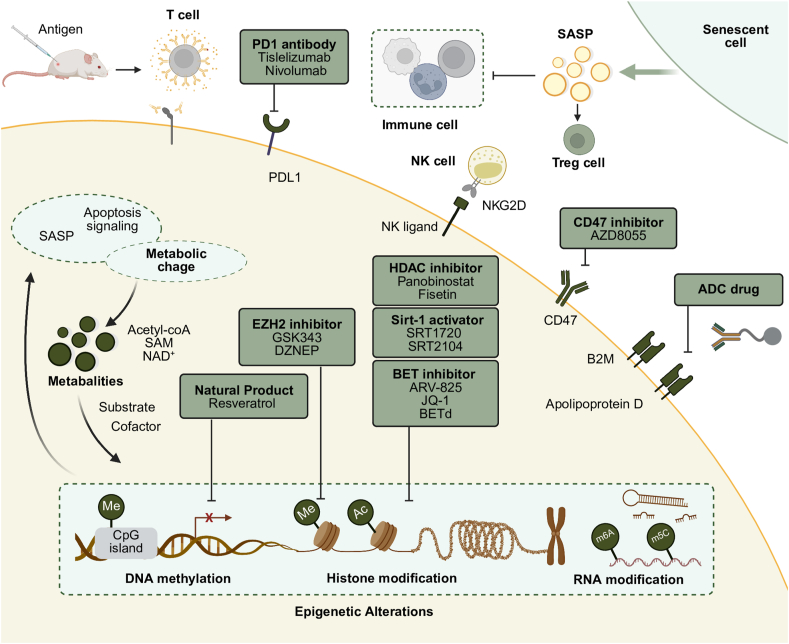
Senolytic drugs clear senescent cells by regulating epigenetics and immune surveillance. The intracellular diagram represents that senolytic drugs clear senescent cells by regulating DNA methylation, histone modification, and RNA modification. The extracellular diagram represents immunotherapy and senolytic agents for clearing senescent cells.

Several other anti-tumor immunotherapies have also been developed for the clearance of senescent cells, such as CAR-T therapy. Anti-urokinase type plasminogen activator receptor CAR-T cell therapy can successfully eliminate senescent cells in immunodeficient mice^249^^,^^250^. Vaccine-based strategies have also been exploited for the therapy of age-associated disorders. After inoculation with the transmembrane glycoprotein NMB vaccine, the number of senescent cells in the mouse model of atherosclerosis decreased, and the median life span of the mouse model of premature aging was prolonged^251^. Vaccination with CD153 vaccine can also reduce the number of aging T cells and prevent the accumulation of fat aging T cells^252^. Finally, the senescent tumor cells or the extract of senescent tumor cells could be used as a cancer vaccine by regulating the immunity. In addition, NK cells have an anti-aging function, which can recognize the receptors and ligands on the surface of aging cells and induce their death^253^. NK receptor NKG2D recognizes the ligand expressed on aging hepatic stellate cells and induces their death through the exocytosis of perforin^254^. In addition, Ouabain can eliminate aging cells by reducing the level of local inflammation and immune infiltration, and may have a synthetic lethal interaction with RAS to exert anti-tumor activity^255^. Therefore, the therapy of enhancing immune-mediated cell clearance rate will also be a potential strategy for the treatment of cancer in the future (Table 4, Fig. 5).

**Table 4:** The mechanisms of drugs that regulate epigenetics and the immune system of senescent cells and tumor cells.

Hallmark	The active compd.	Mechanism as an anti-tumor drug	Mechanism as a senolytic/senomorphic drug	Application in senotherapeutics	Ref.
Epigenetic reprogramming	Panobinostat	Inhibiting histone deacetylation to induce proliferation arrest and apoptosis of tumor cells	Selectively inducing chemotherapy-induced senescent tumor cell death by decreasing Bcl-xL expression and increasing histone H3 acetylation.	Tumor	22
	Fisetin	Inhibiting the proliferation and metastasis of tumor cells by inhibiting HDACs and regulating cell cycle and apoptosis pathways	Selectively removing senescent cells and reducing the release of SASP factors, thereby alleviating inflammation and tissue dysfunction associated with aging	Osteoporosis, osteoarthritis, tumor	164
	Resveratrol	Exerting potent antitumor effects by modulating epigenetic marks, reducing DNA methylation, influencing mitochondrial dynamics, and regulating cell metabolism, apoptosis, and autophagy through the SIRT1–p53 pathway	Delaying the aging process in adipose-derived stem cells by reducing cellular senescence	Neurodegenerative diseases, cardiovascular diseases, and tumors	232
	SRT1720	Inhibiting tumor growth and metastasis by activating SIRT1	Clearing senescent cells by reducing SIRT1-mediated oxidative stress and inflammation	Skin, tumor	234,235
	SRT2104	Preventing tumor-induced muscle and bone wasting by protecting tissue integrity	Inhibiting small extracellular vesicles secreted by senescent cells through activation of SIRT1	Tumor	237.^234^,^235^
	BETd	Blocking the binding of BET proteins to acetylated histones, thereby inhibiting gene expression and exhibiting strong antitumor effects	Attenuating non-homologous end joining and upregulating the expression of autophagic genes, which promotes senolysis in senescent hepatic stellate cells	Tumor	37
	ARV-825	Suppressing the proliferation and inducing apoptosis of T-cell acute lymphoblastic leukemia cells by perturbing the H3K27Ac–Myc pathway and reducing c-Myc protein levels	Selectively killing senescent liver tumor cells, which were induced by the combination of CDK4/6 inhibitor and XPO1 inhibitor	Lung fibrosis, tumor	240
	GSK343	A selective EZH2 (enhancer of zeste homolog 2) inhibitor that reduces H3K27me3 levels, reactivating silenced tumor suppressor genes, and inducing apoptosis	Acting on senescent cells by reversing senescence-associated epigenetic changes, reducing EZH2 levels, and promoting the clearance of senescent cells through apoptosis	Tumor	241
	3-Deazaneplanocin A	An EZH2 degrading agent that reduces EZH2 protein levels, leading to a decrease in H3K27me3 and reactivation of tumor suppressor gene expression	Acting on senescent cells by reversing senescence-associated epigenetic changes, reducing EZH2 levels, and promoting the clearance of senescent cells through apoptosis	Tumor	242
Immune surveillance	Programmed cell death protein 1 antibodies	Clearing tumor cells by immune regulation	Facilitating the clearance of ligand programmed death-ligand 1^+^ senescent cells in an activated CD8^+^ T cell-dependent manner	Nonalcoholic steatohepatitis, tumor	17
	CD47-targeting drugs (AZD8055)	By restoring the phagocytic function of the immune system, it can effectively eliminate tumor cells	Eliminating senescent cells and maintaining tissue homeostasis by inhibiting the SIRP*α*–CD47–SHP-1 axis	Tumor	246
	NK cells	Recognizing the ligand expressed on aging hepatic stellate cells, which induces their death through the exocytosis of perforin	Recognizing the receptors and ligands on the surface of aging cells and inducing their death	Liver fibrosis, IVD, tumor	254
	ADCs (*β*2-microglobulin)	ADC coupled with duocormycin *via* a cleavable enzyme linker was designed against the B2M protein can clear 35% of senescent cells in cancer cell lines	Clearing 35% of senescent cells in cancer cell lines	Pulmonary emphysema, tumor	247
	ADCs (apolipoprotein D antibodies)	Having been frequently utilized in the design of anti-tumor ADCs	Coupling with the pyrrolo benzodiazepine drug PBD in the elderly mouse model selectively eliminated the senescent skin fibroblasts	Skin, tumor	248
	CAR-T therapy	Having been exploited for the treatment of hematologic malignancies	Anti-urokinase type plasminogen activator receptor CAR-T cell therapy can successfully eliminate senescent cells in immunodeficient mice	Tumor	249,250
	Vaccine	Having been exploited for the treatment of various cancers	After inoculation with the transmembrane glycoprotein NMB vaccine, the number of senescent cells in the mouse model of atherosclerosis decreased	Atherosclerosis, tumor	251

## 6 Further discovery and future challenge of senolytic drugs

In recent years, numerous senolytic drugs have been identified and demonstrated to exhibit therapeutic benefits across various preclinical disease models, including kidney and liver diseases^256^^,^^257^, neurodegenerative diseases^258^, cardiovascular diseases^259^ , and cancer^260^. Furthermore, several of these senolytic drugs have advanced to clinical trials. For instance, the first senolytic drug combination, dasatinib and quercetin, has been extensively tested in multiple disease models and is currently being evaluated in clinical trials for conditions such as idiopathic pulmonary fibrosis, chronic kidney disease, and diabetes-related complications^261^. Preliminary findings suggest that this combination may decelerate the progression of certain age-related diseases and improve overall health outcomes^262^. Presently, clinical trials are also investigating the senolytic properties of fisetin, particularly its potential in treating osteoarthritis, Alzheimer's disease, and other age-related conditions. However, further research is necessary to confirm its efficacy and safety in human populations^34^. Similarly, UBX1325, a Bcl-xL inhibitor developed by Unity Biotechnology for the treatment of retinal diseases, is currently undergoing clinical trials for diabetic macular edema and age-related macular degeneration. This drug shows promising potential in reducing the burden of senescent cells within the retina, thereby mitigating vision loss^55^. Piperlongumine, a natural compound extracted from long pepper, has also exhibited anti-aging properties in animal models and is currently under clinical investigation to evaluate its therapeutic potential in humans^263^. Other clinical studies on senolytic drugs are summarized in Table 5.

**Table 5:** The senolytic drugs in clinical trials.

Drug	Class	Diseases	Phase	NCT number
Dasatinib + quercetin	Tyrosine kinase inhibitor/natural product	Idiopathic pulmonary fibrosis, haematopoietic stem cell transplant survivors study, Alzheimer's, diabetic chronic kidney disease, childhood cancer survivors, first degree relatives of type II diabetics, age-related osteoporosis, aging, head and neck squamous cell carcinomas, breast cancer, liver fibrosis, mental disorder	II	NCT05422885
Fisetin	Natural product	Osteoarthritis, frailty, COVID-19, vascular function, peripheral arterial disease, carpal tunnel syndrome, breast cancer,	IV	NCT06819254
ABT-263	Bcl-2 inhibitor	Idiopathic pulmonary fibrosis, myelofibrosis, leukemia, solid tumors, breast cancer, high grade serous carcinoma	III	NCT04472598
ABT737	Bcl-2 inhibitor	Ovarian cancer	Ex vivo	NCT01440504
UBX1325	Bcl-xL inhibitor	Macular degeneration, diabetic macular edema	II	NCT05275205
UBX0101	P53-MDM2 inhibitor	Osteoarthritis	II	NCT04129944
RG-7112	P53-MDM2 inhibitor	Hematologic neoplasms, advanced solid tumor	I	NCT00559533
17-DMAG	Hsp90 inhibitor	Lymphoma, solid tumor, leukemia	I	NCT00089271
DT2216	Bcl-xL PROTAC	Solid tumor, hematologic malignancy, childhood fibrolamellar carcinoma	II	NCT06620302
AZD8055	mTOR inhibitor	Hepatocellular carcinoma, solid tumors, gliomas	I	NCT01316809
Chloroquine	TLRs inhibitor	Glioblastoma, malaria, COVID-19, proliferative nephritis, pancreatic cancer, autoimmune hepatitis, osteoarthritis	IV	NCT00805519
2-Deoxy-d-Glucose	Glycolysis inhibitor	Acute nasopharyngitis, non-small cell lung cancer, diabetes mellitus, gastroparesis, idiopathic dilated cardiomyopathy, prostate cancer, prostate cancer, epilepsy	II	NCT00633087
Resveratrol	Natural product	Osteoarthritis, type 2 diabetes mellitus, colon cancer, liver cancer	IV	NCT02905799
MitoQ	CoQ10 antioxidant	Peripheral arterial disease, aging, fatigue, multiple sclerosis, multiple sclerosis, multiple sclerosis, non-alcoholic fatty liver disease, chronic obstructive pulmonary disease, chronic kidney disease	IV	NCT02364648
Tamoxifen	Estrogen receptor modulator	Breast cancer, diabetes, cardiovascular diseases, duchenne muscular dystrophy	IV	NCT0053777
Metformin	Glucose metabolism modulator	Diabetes, cancer, cardiovascular disease, cognitive impairment	IV	NCT05840068
Dichloroacetate	PDK inhibitor	Pulmonary hypertension, glioblastoma, squamous cell carcinoma of the head and neck, breast cancer, lung cancer, adiposity, acute ischemic stroke	IV	NCT03413202
Orlistat	Fatty acid synthase inhibitor	Obesity, lung adenocarcinoma, nonalcoholic steatohepatitis, diabetes, heart diseases	IV	NCT00752726
Spermidine	Autophagy inducer	Hypertension, heart failure, ischemic heart disease	IV	NCT06792916
Rapamycin	mTOR inhibitor	Advanced solid tumors, diabetes mellitus, cardiovascular disease, cognitive impairment	IV	NCT03793751
Ruxolitinib	JAK inhibitor	Advanced solid tumors, hematologic malignancy, myelofibrosis	IV	NCT02386800
Tocilizumab	TNF inhibitor	Advanced solid tumors, rheumatoid arthritis, hematologic malignancy, diabetes	IV	NCT01331837
Anakinra	IL-1 inhibitor	Kawasaki disease, neuroinflammatory response, rheumatoid arthritis, advanced malignant neoplasm, diabetes	IV	NCT02236481
Xilonix	Anti-IL-1	Colorectal cancer, pancreatic cancer	III	NCT01767857
Infliximab	Anti-TNF*α*	Ulcerative colitis, rheumatoid arthritis, Crohn's disease, inflammatory bowel diseases, diabetes, cognitive dysfunction	IV	NCT06136546
Etanercept	TNF inhibitor	Advanced solid tumors, hematologic malignancy, rheumatoid arthritis, Alzheimer's disease, diabetes,	IV	NCT01716637
Rutin	Natural product	Diabetes, osteoarthritis, renal disease, chronic venous insufficiency	IV	NCT06579482
Panobinostat	Histone deacetylase	Advanced solid tumors, hematologic malignancy, primary myelofibrosis	IV	NCT02386800

Although these promising studies underscore the significant potential of senolytic drugs in treating age-related diseases, clinical research in this field faces several challenges. One significant issue is the toxicity commonly associated with repurposed anti-tumor drugs, such as navitoclax, a Bcl-2 family inhibitor. Although effective, its severe side effects restrict its use, especially in vulnerable populations like the elderly or immunocompromised patients^161^. While senolytic drugs are typically administered intermittently, in a ‘hit-and-run’ manner, which can mitigate some side effects, their use is still primarily confined to elderly individuals or those with underlying health conditions^19^. In addition, developing senolytic drugs capable of precisely targeting senescent cells could be a potential strategy to reduce their side effects. For instance, a research team has developed a novel senolytic prodrug molecule, KSL0608-Se, which can exert its therapeutic effects upon light activation after accurately anchoring to senescent cells. This approach achieves single-cell resolution in the precise identification and elimination of senescent cells, effectively overcoming the issue of “off-target” effects^264^. Consequently, future research must strive to balance drug efficacy with safety, focusing on reducing toxicity and enhancing drug specificity.

The second challenge arises from the heterogeneity of senescent cells, which impacts context-dependent characteristics similar to tumor cells. These cells can be induced by a variety of stimuli, yet the specific markers common to all senescent cells remain poorly defined. Moreover, even cells induced by the same trigger can exhibit different properties^265^. This heterogeneity was initially discovered because senescent cells show varying susceptibility to different senolytic drugs. For instance, dasatinib is particularly effective against senescent preadipocytes, while quercetin is more potent against senescent endothelial cells^31^. Both fisetin and navitoclax are proficient in eliminating senescent fibroblasts and endothelial cells but are less effective against senescent preadipocytes^57^^,^^71^. Consequently, the development of senolytic therapies must account for the heterogeneity of senescent cells, and personalized senolytic treatment, akin to personalized cancer therapies, is emerging as a potential future direction. However, there is no systematic study on senescent heterogeneity, which is context-dependent and depends on the patient's age, sex, tissue location, pathological state, and microenvironment. Therefore, a large number of clinical samples from different ages, genders, disease types, and tissues should be collected and analyzed by RNA sequencing to establish a systematic senescence-related gene expression, which will not only help to find new senescence markers but also guide the selection of appropriate senolytic drugs based on different senescence types.

Thirdly, the limitations of current *in vitro* and *in vivo* models impede the development of senolytic drugs. Current *in vitro* models are typically based on cells induced by specific triggers. However, due to the heterogeneity of senescent cells, no standardized protocol exists for selecting cell types, triggers, or the degree of induction. Furthermore, verifying senescent phenotypes remains a complex and often confusing process. The development of *in vivo* models also faces significant challenges. Existing animal models, such as those involving accelerated aging, gene editing of aging-related genes, or models using organisms like *Drosophila melanogaster*, *Caenorhabditis elegans*, *Danio rerio*, and mini-pigs, fail to fully recapitulate the pathological states of aging-related diseases^266^. Additionally, the physiological differences between these models and humans are substantial, necessitating the creation of more advanced and comprehensive *in vivo* models. Therefore, the appropriate model to induce senescence is important for the exploitation of senolytic drugs. First, further studies on the pathological mechanisms and developmental characteristics of aging are necessary to inform suitable aging induction methods. Second, it is a potential strategy to develop human organoids for the construction of aging models, which is more consistent with the actual situation of aging in humans.

Finally, the methodologies for discovering senolytic drugs are currently limited. The first-generation senolytic drugs were identified using hypothesis-driven, mechanism-based approaches, while second-generation senolytic drugs have been discovered through high-throughput screening and other conventional drug discovery methods^19^. However, these approaches are somewhat empirical and lack reproducibility, highlighting the need for the development of novel drug discovery strategies. Repurposing anticancer drugs as senolytic drugs is a promising strategy with considerable feasibility. Given the shared molecular mechanisms between senescent cells and cancer cells, such as anti-apoptotic pathways (*e*.*g*., Bcl-2 family) and metabolic alterations (*e*.*g*., glycolysis), high-throughput screening can be employed to identify compounds from existing anticancer drug libraries that target these pathways and exhibit senolytic potential. This approach enhances the specificity of drug library screening. Additionally, the extensive research on the toxicity, target activity, and pharmacological properties of anticancer drugs provides a solid theoretical foundation for the development of senolytic agents. Furthermore, the development strategies for anticancer drugs offer valuable guidance for senolytic drug discovery. For instance, both senescent cells and cancer cells exhibit significant heterogeneity, and sequencing technologies can be utilized to identify genetic mutations and molecular signatures in senescent cells, enabling the design of personalized treatment regimens. Combining drugs with different mechanisms of action can reduce drug resistance and improve therapeutic efficacy. Novel anticancer therapies, such as photodynamic therapy, immunotherapy, nanodelivery systems, and electric field therapy, can also be adapted for the clearance of senescent cells. Therefore, in this review, we have generalized the meta-hallmarks of senescent cells and tumor cells, summarized the existing senolytic drugs based on their regulation of these meta-hallmarks, and discussed their underlying molecular mechanisms. These insights offer fresh insights into the repurposing of anti-tumor drugs as senolytic drugs and inspire new strategies for the discovery of novel senolytic therapies.

## 7 Conclusions

In light of the increasing burden of aging and the profound impact of age-related diseases on human health, understanding the mechanisms of aging is of paramount importance. As a key hallmark of aging, senescent cells exhibit several similarities to tumor cells, including apoptosis resistance, metabolic reprogramming, epigenetic alterations, changes in secretory phenotypes, and immune evasion. In this review, we have summarized these similar features between senescent cells and tumor cells, aiming to deepen the understanding of the mechanisms underlying cellular senescence through the lens of these similar biomarkers. Senolytic drugs, which selectively eliminate senescent cells, have demonstrated significant therapeutic potential across various preclinical disease models. Notably, the majority of these senolytic drugs are derived from anti-tumor drugs, and their mechanisms of action in clearing senescent cells closely align with those used to target tumor cells. Accordingly, we have categorized and discussed existing senolytic drugs based on their similar biomarkers with tumor cells and explored their molecular mechanisms in depth. Furthermore, we have discussed the challenges in senolytic drug discovery, along with future directions. This review offers new insights into the development of senolytic drugs, emphasizing the repurposing of anti-tumor drugs or the adoption of anti-tumor drug development strategies.

## Author contributions

Wei Liu: Conceptualization, Writing-original draft, Investigation; Bo Fan: Writing-original draft; Te Fang: Writing-original draft; Hongyao Li: Writing-original draft; Jin Zhang: Writing-review & editing, Validation; Bo Liu: Supervision, Resources, Funding acquisition. Zhiyu Liu: Conceptualization, Supervision.

## Conflicts of interest

The authors declare no conflicts of interest.
